# High-resolution structure of a fish aquaporin reveals a novel extracellular fold

**DOI:** 10.26508/lsa.202201491

**Published:** 2022-10-13

**Authors:** Jiao Zeng, Florian Schmitz, Simon Isaksson, Jessica Glas, Olivia Arbab, Martin Andersson, Kristina Sundell, Leif A Eriksson, Kunchithapadam Swaminathan, Susanna Törnroth-Horsefield, Kristina Hedfalk

**Affiliations:** 1 Department of Biological Sciences, National University of Singapore, Queenstown, Singapore; 2 Department and Chemistry and Molecular Biology, Gothenburg University, Göteborg, Sweden; 3 Department of Chemistry and Chemical Engineering, Applied Surface Chemistry, Chalmers University of Technology, Gothenburg, Sweden; 4 Department of Biology and Environmental Sciences, Gothenburg University, Göteborg, Sweden; 5 Department of Biochemistry and Structural Biology, Centre for Molecular Protein Science, Lund University, Lund, Sweden

## Abstract

Aquaporins are protein channels embedded in the lipid bilayer in cells from all organisms on earth that are crucial for water homeostasis. In fish, aquaporins are believed to be important for osmoregulation; however, the molecular mechanism behind this is poorly understood. Here, we present the first structural and functional characterization of a fish aquaporin; cpAQP1aa from the fresh water fish climbing perch (*Anabas testudineus*), a species that is of high osmoregulatory interest because of its ability to spend time in seawater and on land. These studies show that cpAQP1aa is a water-specific aquaporin with a unique fold on the extracellular side that results in a constriction region. Functional analysis combined with molecular dynamic simulations suggests that phosphorylation at two sites causes structural perturbations in this region that may have implications for channel gating from the extracellular side.

## Abstract (precis)

The structural and functional characterization of a fish AQP reveals a water-specific AQP with unique structural features that may have implications for channel gating in response to osmotic changes.

## Introduction

**Figure 1.**
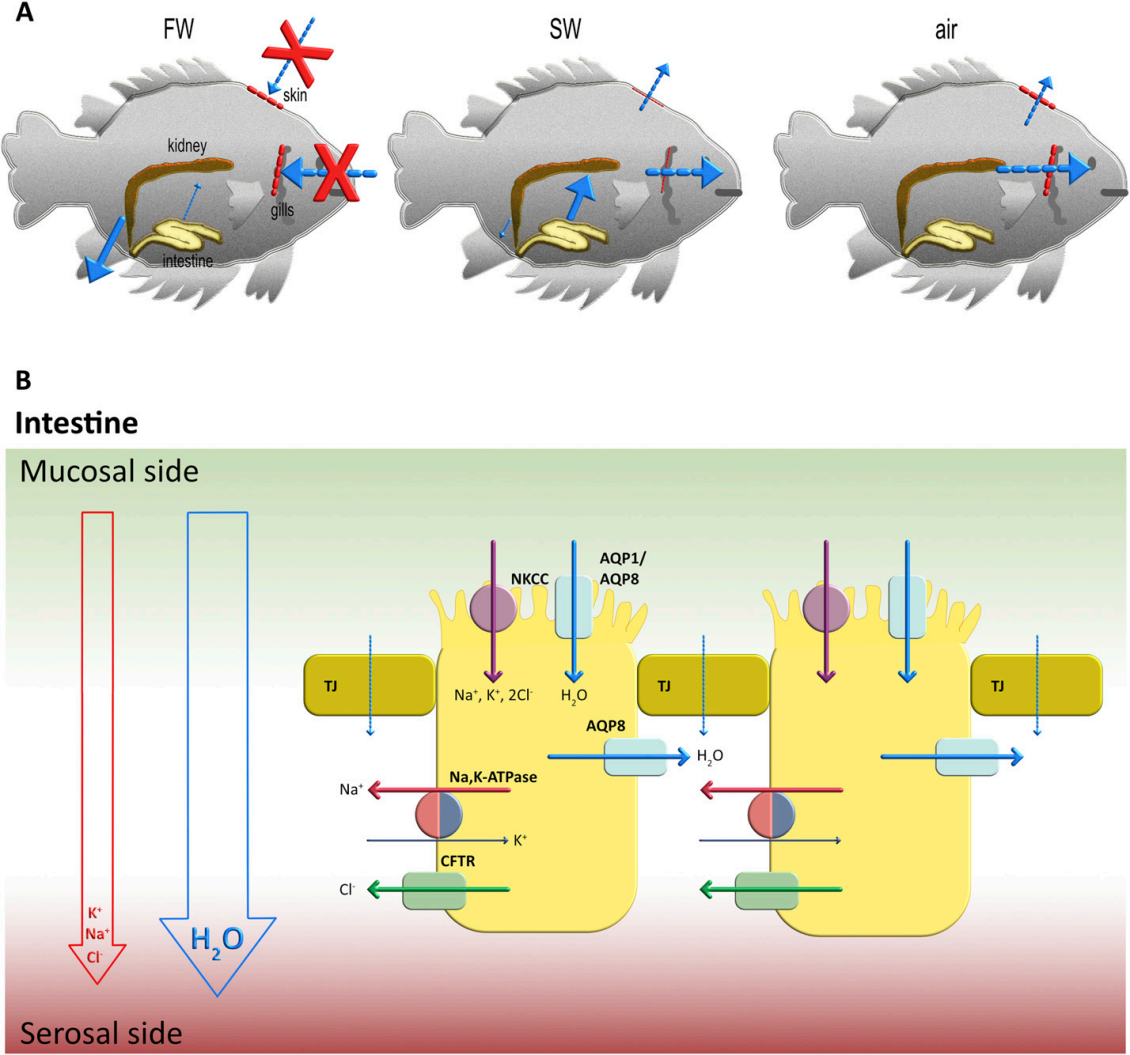
Water passage in climbing perch and water flow in the fish intestine. **(A)** Schematic illustration of the assumed main water flow for climbing perch in fresh water, seawater (SW), and air, respectively, where the size of the arrow corresponds to the water flow and the tissue barrier is indicated by a dashed red line. In fresh water, *Anabas testudineus* is a hyperosmotic osmoregulator, aiming to minimize the diffusional inflow of water across the gills and producing dilute urine to get rid of excess water. In SW, on the other hand, *A. testudineus* will acclimate and become a hypoosmotic osmoregulator with the reverse problem with water diffusing out across all epithelia. This is counteracted by an ion-coupled water uptake across the intestine and a decreased glomerular filtration rate and urine production. During terrestrial exposure, *A. testudineus* must reduce water loss from the large surface areas constituted especially by the gills but also by the skin. The intestinal water absorption is also likely to be reduced as that is dependent on drinking, which ceases in air. **(B)** During SW acclimation, a total water flow is directed from the mucosal to the serosal side of the intestine driven by an ionic gradient of mainly K^+^, Na^+^, and Cl^−^, involving ion transport via NKCC, Na,K-ATPase, and CFTR. In the SW trout, the increased intestinal water absorption is facilitated by aquaporin isoforms in the apical (AQP1a and AQP8ab) and basolateral (AQP8ab) membranes, respectively (reviewed in Madsen, 2015). The figure shows two enterocytes separated by tight junctions (TJ).

Osmoregulation is a major challenge for fish because they are in direct contact with water and continuously need to compensate for passive water loss or uptake, depending on the environment (Evans, 2008). Among the fish species, the osmoregulation of the climbing perch (*Anabas testudineus*) is of particularly high interest because of being extremely adaptable to environmental changes. *A. testudineus* is a fresh water teleost that (i) can acclimate from fresh water to seawater (SW) over 7 d in a progressive manner (Chang et al, 2007), (ii) is able to live on land for up to 6 d and use air by breathing through the “labyrinth organ,” a special accessory respiratory organ (ABO) located in the upper part of the gill chambers (Davenport & Abdulmatin, 1990), and (iii) is capable of active ammonia excretion during emersion (Tay et al, 2006; Ip et al, 2013) (Fig 1A). For these reasons, *A. testudineus* provides an attractive model organism for studying the mechanisms of water homeostasis and ammonia excretion in fish.

Although the ionic osmoregulatory mechanisms and the structural organization of both gill and the intestinal epithelia are very well understood (Grosell, 2010; Sundell & Sundh, 2012; Lema et al, 2018), the mechanism for controlling water flow through aquaporins (AQPs) in various fish tissues and organs are less well studied (Sundell & Sundh, 2012; Madsen et al, 2015). AQPs are membrane-integral water channels that are present in all organisms where they facilitate passive flow of water across the membrane as a direct response to the osmotic pressure. AQPs in fish were first identified in 2000 and are distributed in the skin, gill, intestine, and kidney (Cutler, 2000). Interestingly, there seems to be a connection between the development of the fish and the diversity of AQPs produced, reflecting the trends of evolution (Tingaud-Sequeira et al, 2008), as illustrated by 18 and 42 AQP members having been identified in the model organism zebrafish *Danio rerio* (Tingaud-Sequeira et al, 2008) and the Atlantic salmon (*Salmon salar*) (Madsen et al, 2015), respectively. Furthermore, expression levels vary with changes in salinity supporting the involvement of AQPs in fish osmoregulation (Guo et al, 2017). Most of our current understanding of the osmoregulatory role of AQPs in fish concerns the fish intestine where they are found in both the apical and basolateral membranes of intestinal epithelial cells, mediating transcellular water flow and intestinal water uptake in SW (Fig 1B) (Fischbarg, 2010; Madsen et al, 2015). Investigation of numerous fish species reveals that AQPs are up-regulated in the intestine as a response to transfer to SW, indirectly supporting their involvement in a general mechanism for osmoregulation and water absorption (Sundell & Sundh, 2012; Madsen et al, 2015). The presence of AQPs in gills and skin suggest that they are also involved in regulated transmembrane water flow in these organs, but little is known about the osmoregulatory role of AQPs found in tissues and organs that are in direct contact with the surrounding (Madsen et al, 2015).

**Figure 2.**
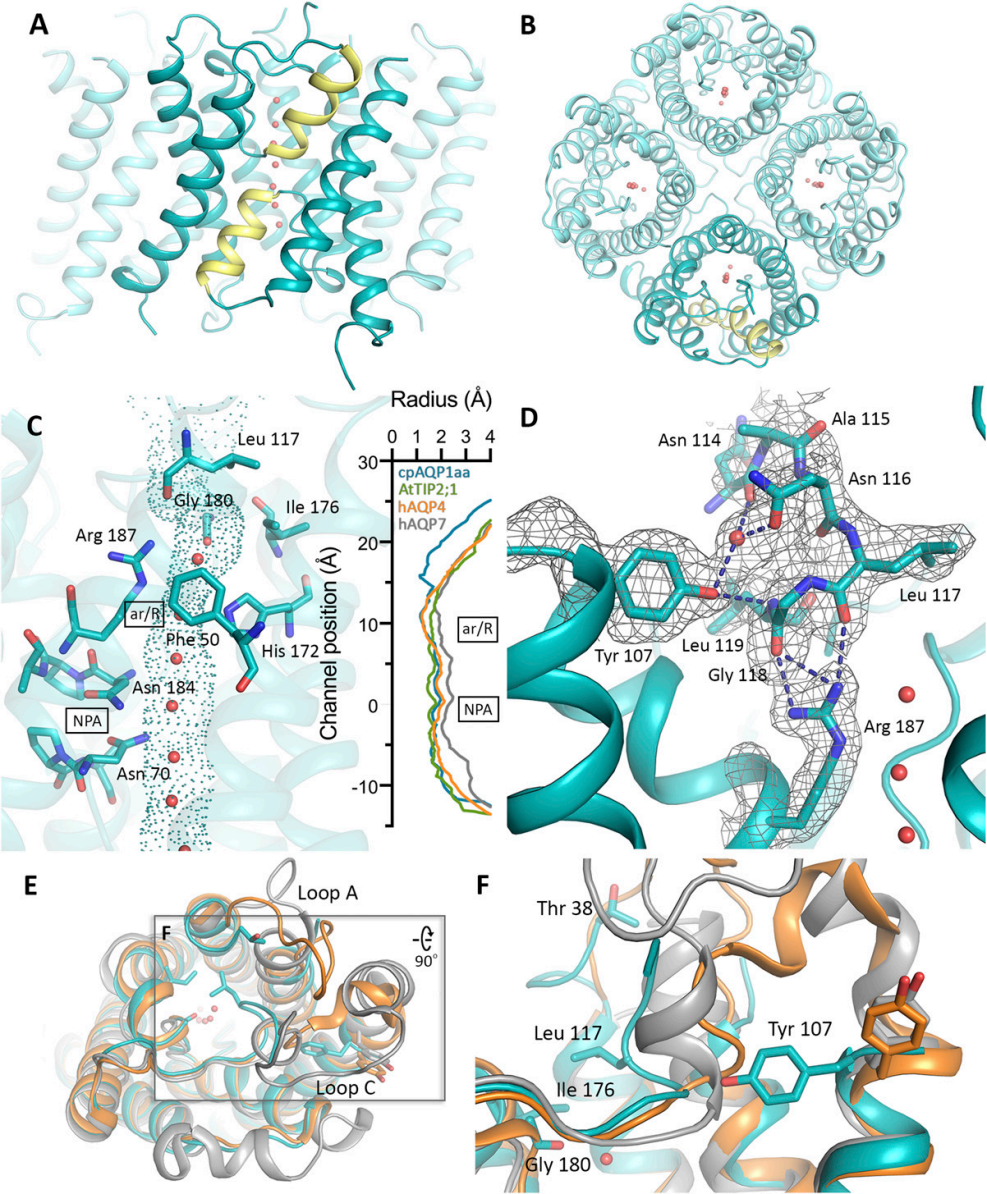
Crystal structure of cpAQP1aa. **(A, B)** The cpAQP1aa tetramer viewed (A) parallel to the membrane and (B) from the extracellular side. The two half-helices forming the seventh pseudo-transmembrane segment are colored yellow in the frontmost monomer. Water molecules inside the channel are showed as red spheres. **(C)** HOLE analysis of the water-conducting channel with the channel dimensions visualized as dots. Residues in the aromatic-arginine (ar/R) and NPA regions as well as the extracellular vestibule are shown in stick representation. The graph shows a comparison between the channel profiles of cpAQP1aa (teal), human AQP4 (orange, PDB code 3GD8), human AQP7 (gray, PDB code 6QZI), and *Arabidopsis* TIP2;1 (green, PDB code 5I32). The zero channel position corresponds to the midpoint between the two NPA motifs. For cpAQP1aa, the channel is significantly narrower on the extracellular side of the ar/R region with a channel radius of 1.1 Å around Leu 117 (∼16 Å from the NPA region). **(D)** Close-up view of the hydrogen-bonding network involving Tyr 107, Arg 187, and residues 114–119 that stabilizes the unique conformation of loop C in cpAQP1aa. Hydrogen bonds are depicted as blue dashed lines. Electron density shown as gray mesh represents the 2FoFc-map contoured at 1.0 σ. **(E)** Structural overlay of cpAQP1aa (teal), human AQP4 (orange), and human AQP7 (gray), viewed from the extracellular side, showing that the main structural differences are found in loops A and C. Residues in the cpAQP1aa extracellular constriction region and putative phosphorylation site at Thr 38 in loop A are shown in stick representation. Water molecules in the channel are shown as red spheres. **(E, F)** Zoom-in on boxed region in (E), viewed from the side of the membrane (90 degree rotation), showing significant structural differences in loop C between the three overlaid structures. In cpAQP1aa, the side chain of Tyr 107 has flipped compared with AQP4 and AQP7, pushing loop C further into the extracellular vestibule closer to the channel opening.

AQPs are commonly divided into three subgroups: orthodox AQPs, permeable to water only; aquaglyceroporins that also permit flow of larger solutes such as glycerol and urea; and superaquaporins with unknown solute specificity (Ishibashi et al, 2009). In addition, some AQPs have been shown to allow passage of gas, most notably ammonia and carbon dioxide, and a selection of the AQP isoforms allow flow of hydrogen peroxide as part of the cellular redox signaling (Nordzieke & Medrano-Fernandez, 2018). High-resolution structures of various AQP homologs have revealed a common homotetrameric fold, with each monomer constituting a functional pore and comprising six transmembrane helices. In addition, two of the connecting loops form half membrane–spanning helices, each containing one copy of the highly conserved NPA (asparagine-proline-alanine) motif (Ozu et al, 2018) (Fig 2). Within the pore, the diameter and polarity of the aromatic/arginine (ar/R) motif located near the extracellular vestibule determine the substrate specificity of the AQP channel, with the pore diameter being restricted to ∼3 Å in water-specific AQPs and typically being 1 Å wider in aquaglyceroporins (Fu et al, 2000; Sui et al, 2001). High-resolution structural studies have also explained how the NPA- and ar/R motifs together prevent protons being conducted along the single file of water molecules inside the pore via the Grotthuss mechanism (Tornroth-Horsefield et al, 2010; Eriksson et al, 2013). Eukaryotes have evolved to posttranslationally regulate water flow through AQPs by gating or trafficking in response to environmental or cellular signals. Gating has been proposed for several eukaryotic aquaporin homologs and includes restricting the channel radius by larger conformational changes (capping) or small side-chain movements (pinching) (Hedfalk et al, 2006). Structural studies have resulted in detailed gating mechanisms to be proposed for mammalian AQP0 (Gonen et al, 2004; Reichow et al, 2013), SoPIP2;1 from plant (Tornroth-Horsefield et al, 2006; Nyblom et al, 2009; Frick et al, 2013a, 2013b) and Aqy1 (Fischer et al, 2009) from yeast, involving triggers such as pH (AQP0 and SoPIP2;1), phosphorylation (SoPIP2;1 and Aqy1), Ca^2+^-binding (SoPIP2;1), protein–protein interactions (AQP0), and mechanosensitivity (Aqy1).

**Figure S1.**
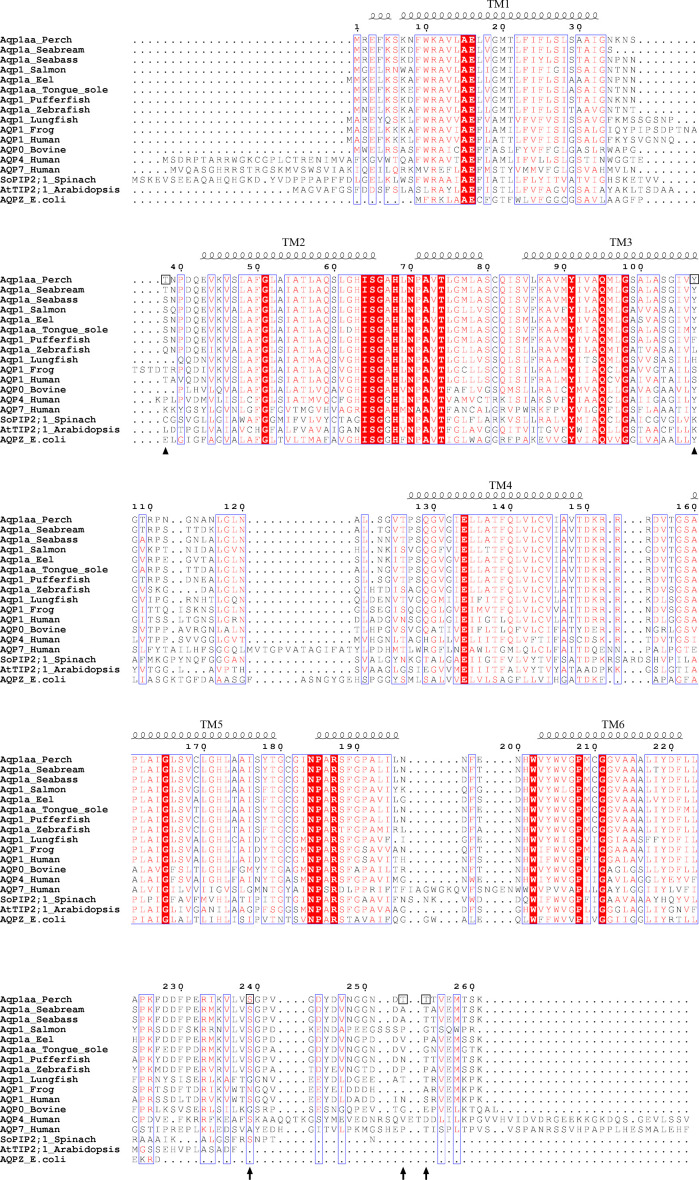
Sequence alignment of selected aquaporin homologs from various species. Strictly conserved residues are rendered with white characters on a red background in the column. Well-conserved residues (>70% similarity) are rendered as red characters in the column. Specific amino acids of importance for cpAQP1aa regulation or modification are marked by black boxes and highlighted by arrows; namely, T38 and Y107 involved in channel gating and the three confirmed C-terminal phosphorylation sites. Sequence alignment was performed using Clustal Omega, EMBL-EBI (https://www.ebi.ac.uk/Tools/msa/clustalo/), and the figure was made with ESPript 3.0 (http://espript.ibcp.fr/ESPript/ESPript/).

To shed light on the osmoregulatory role of AQPs in fish, we have structurally and functionally characterized AQP1aa from *A. testudineus* (cpAQP1aa), a homolog of mammalian AQP1 and the only characterized AQP from this species to date (Ip et al, 2013) (Fig S1). Our work provides the first structural and functional characterization of a fish AQP and shows that cpAQP1aa is a water-specific AQP with a unique pore narrowing at the extracellular side. Mutational analysis combined with molecular dynamic (MD) simulations suggest that perturbations of this region may lead to conformational changes that could control the water flow through cpAQP1aa, the implications of which in relation to a possible extracellular gating mechanism is discussed. This study contributes significantly to our understanding of the role of AQPs in fish and suggests new intriguing possibilities for the variation in molecular mechanisms that control water flow through the AQP family of proteins.

## Results

### High-resolution structure of cpAQP1aa reveals a novel fold on the extracellular side

Because flexible termini are known to hamper crystallization of AQPs, C-terminally truncated cpAQP1aa (cpAQP1aa-243, where residues 243–261 were deleted) was produced in *Pichia pastoris*, purified, and crystallized. The structure was solved to 1.9 Å resolution by molecular replacement using a homology model of cpAQP1aa based on the crystal structure of bovine AQP1 (PDB code 1J4N) (Sui et al, 2001) (Table S1). The overall structure of cpAQP1aa shares most of the common AQP structural features; assembling as a homotetramer with each monomer forming a functional pore and consisting of six transmembrane α-helices (1–6) and five connecting loops (A-E) (Fig 2A and B). A seventh pseudo-transmembrane segment is formed by the insertion of two half-helices in loops B and E into the pore, each of which contain the AQP signature NPA motif. A single file of water molecules is aligned within the pore of each monomer, indicating that cpAQP1aa is in a functional state. Loops A and C are partly disordered, with residues 34–37 and 108–112, lacking electron density, and these parts have been omitted from the structure. The last residue that could be unambiguously modeled is Lys 226, wherefore its C-terminus is deemed to be disordered beyond this residue.

Table S1 Data collection and refinement statistics of cpAQP1aa.

To explore the features of the cpAQP1aa pore, its shape and dimensions were determined using the program HOLE (Smart et al, 1993) (Fig 2C). Similar to other AQPs, the cpAQP1aa pore is dumbbell-shaped, consisting of open vestibules at both intracellular and extracellular sides, with an elongated narrow pore in between. The pore constricts to a radius of ∼1.2 Å at the ar/R region, formed by residues Arg 187, His 172, Cys 181, and Phe 50, a width and residue composition that is typical for water-specific AQPs (Sui et al, 2001). However, when comparing the channel profile of cpAQP1aa with those of the water-specific channel human AQP4 (Ho et al, 2009), the aquaglyceroporin human AQP7 (de Mare et al, 2020), and the ammonia-permeable TIP2;1 from *Arabidopsis thaliana* (Kirscht et al, 2016), a unique pore narrowing at the extracellular side is identified. This region is defined by a novel conformation of loop C, placing residues 114–117 (cpAQP1aa numbering) closer to the center of the extracellular vestibule. As a result, Leu 117, Ile 176, and Gly 180 form a hydrophobic constriction region with a radius of 1.1 Å, ∼8 Å away from the ar/R region. The unique conformation of loop C is stabilized by a hydrogen-bonding network that involves Tyr 107 in transmembrane helix three, residues 114–119 in loop C, and Arg 187 within the ar/R region (Fig 2D). Specifically, hydrogen bonds are formed between the side chain of Tyr 107 and the backbone nitrogen of Leu 119 as well as the carbonyl oxygen and side chain of Asn 114 and 116, respectively, via a water molecule. In addition, the Arg 187 guanidino group forms hydrogen bonds to the backbone oxygens of Leu 117 and Gly 118.

**Figure S2.**
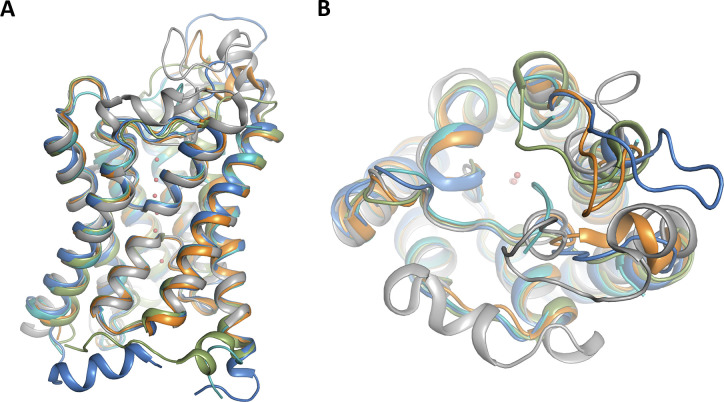
Structural comparison of eukaryotic aquaporins. **(A, B)** Overlay of crystal structures of cpAQP1aa (teal), bovine AQP1 (blue), human AQP4 (orange), human AQP7 (gray), and *Arabidopsis* TIP2;1 (green) viewed (A) parallel to the membrane and (B) from the extracellular side. Water molecules are shown as red spheres.

A structural comparison between cpAQP1aa, human AQP4 (water-specific), and human AQP7 (glycerol-permeable) (Fig 2E) as well as an extended comparison with several other eukaryotic AQPs (Fig S2) shows structural variability in loops A and C, highlighting that these two loops are structurally not well conserved. Although Tyr 107 is conserved in all three AQPs, its side chain is pointing away from the channel in AQP4 and AQP7, resulting in loop C remaining further away from the channel opening (Fig 2F). Furthermore, Leu 117 is highly conserved in vertebrate AQPs, including fish and human (albeit not in human AQP7) (Fig S1), indicating that the extracellular constriction region is not directly related to this residue being present or not. It should be pointed out that despite the restricted pore diameter, this region is not narrow enough to completely exclude water molecules and additional conformational changes would be needed to block water flow.

### The extracellular fold of cpAQP1aa is unaffected by pH

**Figure S3.**
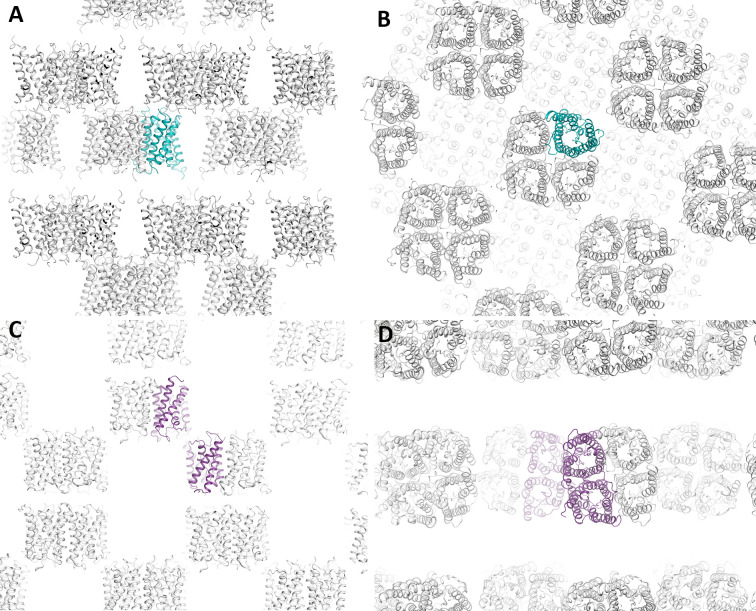
Crystal packing for cpAQP1aa at different pH. Cartoon representation of crystal packing in cpAQP1aa crystals. **(A, B)** Crystal packing in cpAQP1aa grown at pH 7.8. The views differ by a 90° rotation toward the viewer. **(C, D)** same as in (A) and (B) but for cpAQP1aa crystals grown at pH 6.5. The protein molecules corresponding to the asymmetric unit are colored teal (pH 7.8) and purple (pH 6.5).

**Figure S4.**
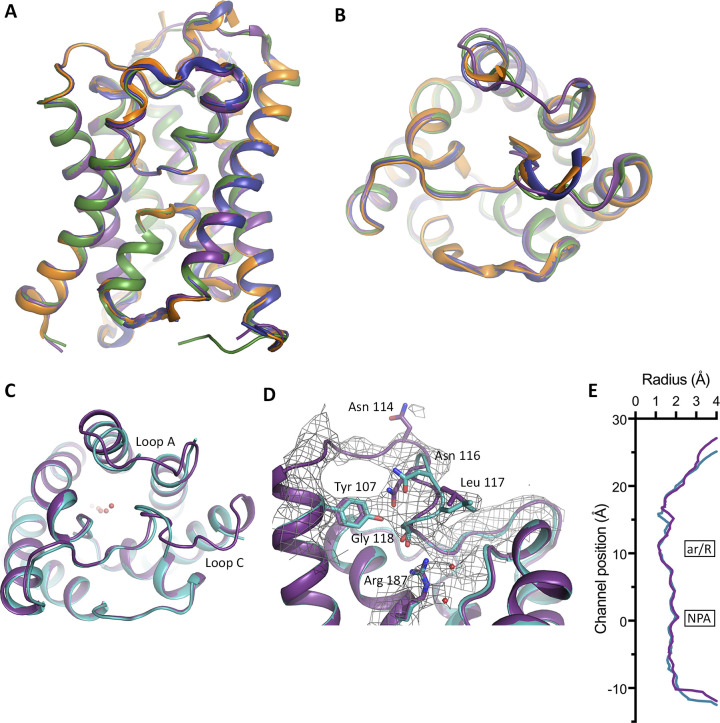
Structure of cpAQP1aa at pH 6.5. **(A, B)** Overlay of the four monomers in the cpAQP1aa tetramer viewed (A) parallel to the membrane and (B) from the extracellular side, displaying only minor structural differences between the monomers. In one monomer only, monomer C (purple) and loops A and C could be built in full. **(C)** Structural overlay of the cpAQP1aa structures at pH 7.8 (teal) and 6.5 (monomer C, purple). The two structures are highly similar but differ in loops A and C. Although these loops could be fully built in monomer C of the low pH structure, they were mostly disordered in all monomers in the high pH structure. **(D)** Zoom-in on the extracellular part of the overlay in (C), showing that despite the differences in loop C, the distal part of the loop occupies a similar position in both structures, including the residues that participate in the hydrogen-bonding network that stabilizes its unique conformation. Electron density (gray mesh) for loop C and Arg 187 in the low pH structure is a composite omit map displayed at 1.0 σ. **(E)** Graph comparing the channel profiles of cpAQP1aa structures at pH 6.5 (monomer C, purple) and 7.8 (teal), as calculated by HOLE. In both structures, a similar constriction can be seen on the extracellular side of the ar/R region. The zero channel position corresponds to the midpoint between the two NPA motifs.

To investigate if the conformation of loop C that is responsible for the cpAQP1aa extracellular constriction region is dependent on pH, we crystallized and solved the structure of cpAQP1aa at a lower pH; pH 6.5 instead of 7.8. The low pH crystals belonged to a different space group, C222_1_ instead of P42_1_2, and diffracted to significantly lower resolution (3.5 Å) (Table S1). The lower diffraction quality is consistent with the differences in crystal packing; the crystals grown at the lower pH have considerably weaker crystal contacts and higher solvent content compared with the crystals at pH 7.8 (Fig S3). Furthermore, in the structure at lower pH, there are four cpAQP1aa monomers in the asymmetric unit compared with one in the structure at higher pH. Because of this, structural differences can be observed between the monomers, most notably the fact that loops A and C could be built in their entirety in one of the monomers (monomer C) (Fig S4A and B).

As seen in Fig S4C and D, the two structures at different pH are very similar, although some minor differences can be seen in loops A and C. Nevertheless, the part of loop C that allows the formation of the extracellular constriction region (residues 114–119) occupies a similar position at both high and low pH. In particular, Tyr 107, Asn 116, Leu 117, Gly 118, and Arg 187 line up well and seem to be able to participate in the same hydrogen-bonding network in both structures (hydrogen bonds cannot be reliably assigned in the low pH structure because of the limited resolution) (Figs 2D and S4D). The channel profiles, as calculated by HOLE, are very similar in both structures (Fig S4E), suggesting that the extracellular hydrophobic constriction region is unaffected by pH.

### cpAQP1aa is a channel for water but not glycerol

**Figure S5.**
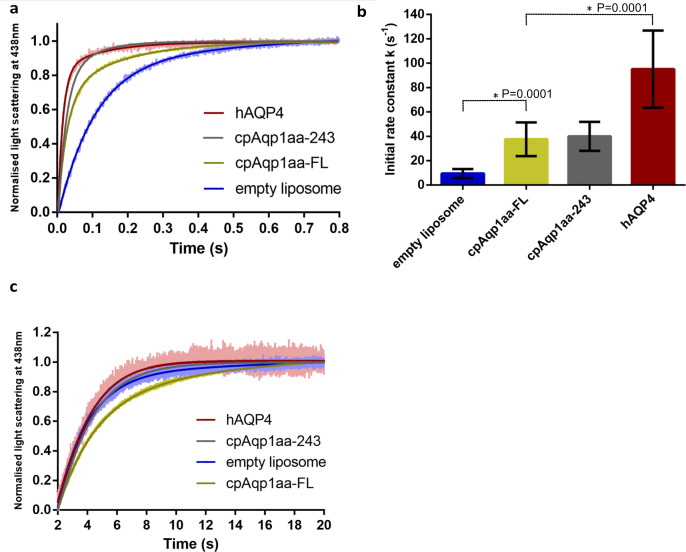
Functional analysis of the cpAQP1aa. **(A)** Representative curves for the water flow of hAQP4 (positive control), cpAQP1aa-243, cpAQP1aa-FL, and empty liposome (negative control), showing that purified protein of both cpAQP1aa-FL and cpAQP1aa-243 are functional. **(B)** Initial rate (i.r.) of the water flow of hAQP4 (positive control), cpAQP1aa-243, cpAQP1aa-FL, and empty liposome (negative control), showing that purified protein of both cpAQP1aa-FL and cpAQP1aa-243 are functional (n = 9–25 ± SD). **(C)** Representative curves for glycerol flow of cpAQP1aa-FL, cpAQP1aa-243, hAQP4, and empty liposome (negative control) (n = 3).

**Figure S6.**
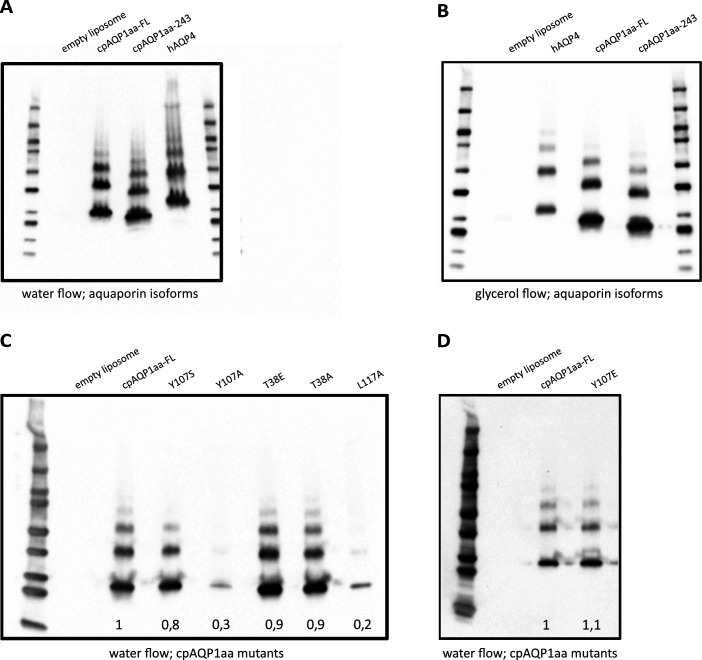
Immunoblots showing liposome reconstitution. **(A)** Immunoblot using the 6× His monoclonal antibody (Takara Clontech) showing typical proteoliposome preps for the empty liposome (negative control), cpAQP1aa-FL, cpAQP1-243, and hAQP4 (positive control) for the functional analysis of the water flow. **(B)** Immunoblot using the 6× His monoclonal antibody (Takara Clontech) showing typical proteoliposome preps for the empty liposome (negative control), hAQP4 (positive control), cpAQP1aa-FL, and cpAQP1-243 for the functional analysis of the glycerol flow. **(C)** Immunoblot using the 6× His monoclonal antibody (Takara Clontech) showing the liposome preps for the empty liposome (negative control), cpAQP1aa-FL, cpAQP1aa-Y107S, cpAQP1aa-Y107A, cpAQP1aa-T38E, cpAQP1aa-T38A, and cpAQP1aa-L117A. The relative protein reconstitution efficiencies are averages from two immunoblots. **(D)** Immunoblot using the 6× His monoclonal antibody (Takara Clontech) showing the liposome preps for the empty liposome (negative control), cpAQP1aa-FL, and cpAQP1aa-Y107E. The relative protein reconstitution efficiencies are averages from four immunoblots.

To verify that the cpAQP1aa produced in *P. pastoris* is a functional water channel, as suggested by the crystal structure, both the full-length protein (cpAQP1aa-FL) and the truncated variant used for crystallization (cpAQP1aa-243) were reconstituted in liposomes and tested for water flow by stopped-flow spectroscopy (Fig S5A). The rate of water flow was compared with that of human AQP4 (hAQP4) and the empty liposomes. The initial rate constants were 37.59 ± 13.84 s^−1^ and 39.97 ± 11.92 s^−1^ for cpAQP1aa-FL and cpAQP1aa-243, respectively (Fig S5B). This is significantly higher than that for the empty liposome (9.49 ± 3.68 s^−1^), showing that both cpAQP1aa constructs are equally permeable to water. However, compared with the initial rate constant for hAQP4 (95.13 ± 31.67 s^−1^), a highly efficient water-specific AQP with a fully open pore, the rate constant of cpAQP1aa is substantially lower. This is in agreement with the cpAQP1aa pore being restricted but not closed, as observed in the crystal structure (Fig 2A and B). As seen in Fig S6A, the liposomes contain similar levels of protein, showing that the differences in water flow rates between hAQP4 and cpAQP1aa are not a result of liposome reconstitution efficiency.

Because many AQPs have broader specificity than water, glycerol permeability was also evaluated. Neither of the reconstituted AQPs, cpAQP1aa-FL, cpAQP1aa-243, or hAQP4, permitted flow of glycerol (Fig S5C). Similarly as for the water flow assay, immunoblot analysis supports that the AQPs are properly reconstituted, with cpAQP1aa being reconstituted at slightly higher levels than hAQP4 (Fig S6B). The lack of glycerol flow through cpAQP1aa is well related to the observed diameter and structure of the pore, which is significantly narrower than the glycerol facilitator human AQP7 (Fig 2C).

### The structural features of the cpAQP1aa pore do not support passage of ammonia

**Figure 3.**
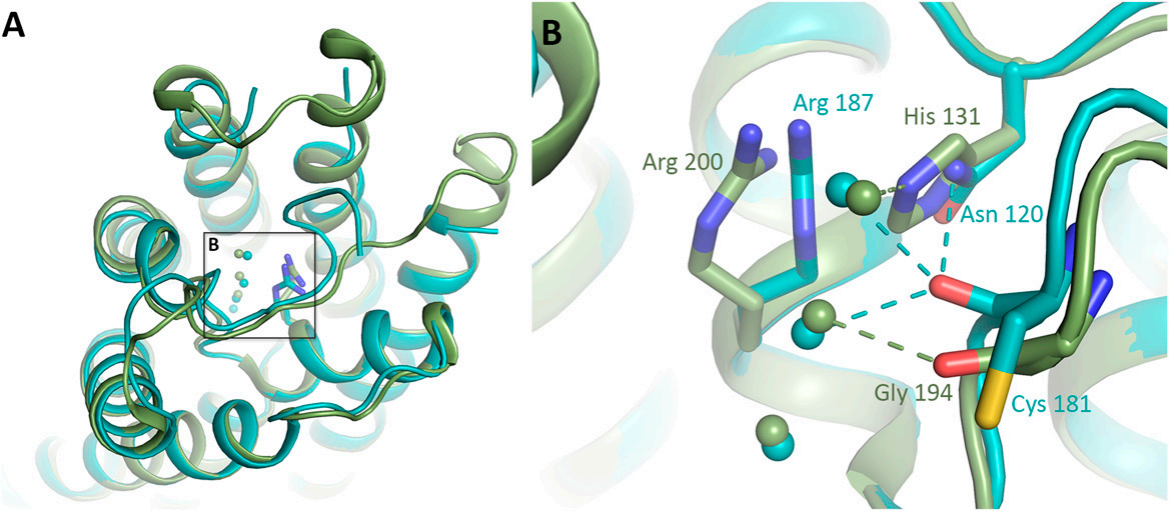
Structural comparison between cpAQP1aa and the ammonia-facilitating aquaporin AtTIP2;1. **(A)** Overlay of cpAQP1aa (teal) and AtTIP2;1 (green) viewed from the extracellular side. The arginine in the ar/R region is shown in stick representation. Water molecules in the channel are shown as spheres of the respective protein color. **(A, B)** Zoom-in on the boxed area in (A), showing the different side-chain orientation of the arginine in the ar/R region and the hydrogen-bonding pattern between water molecules and residues within the channel. Hydrogen bonds are shown as dotted lines in the respective protein color. In cpAQP1aa, the carbonyl position of Cys 181 and its hydrogen bonds to Asn 120 and two water molecules correspond to what is typically seen in water-specific AQPs. In contrast, the carbonyl of Gly 194 in AtTIP2;1 occupies a different position, observed mainly in non–water-specific AQPs, and shows a different hydrogen-bonding pattern.

To explore if the cpAQP1aa pore could permit passage of ammonia, we compared its structural features to those of the ammonia channel PIP2;1 from *A. thaliana* (AtPIP2;1). The ammonia channel AtPIP2;1 has been described as comprising an extended specificity filter, which, together with a deprotonation mechanism provided by His 131, is believed to contribute to ammonia passage, in addition to water (Kirscht et al, 2016). The presence of a histidine in position 131 forces the side chain of the arginine in the ar/R region of AtTIP2;1 to adopt a novel conformation that has not been seen in other AQPs (Fig 3). Furthermore, the ammonia permeability of AtTIP2;1 has been proposed to correlate with the conformation of Gly 194, for which the carbonyl position and hydrogen-bonding pattern is typical for non–water-specific AQPs. When comparing this region in AtTIP2;1 and cpAQP1aa, it is evident that the structure of cpAQP1aa most strongly resembles those of water-specific AQPs. Specifically, the presence of an asparagine (Asn 120 in cpAQP1aa) in the equivalent position to His 131 in AtPIP2;1, the position of the cpAQP1aa Cys 181 carbonyl (equivalent position as Gly 194 in AtTIP2;1), and the hydrogen-bonding pattern between residues and water molecules all point at cpAQP1aa being a water-specific AQP. This suggests that it is rather unlikely that cpAQP1aa plays a major role in ammonia transmembrane passage as previously proposed based on mRNA analysis (Ip et al, 2013).

### Mutational analysis of the extracellular constriction region

**Figure 4.**
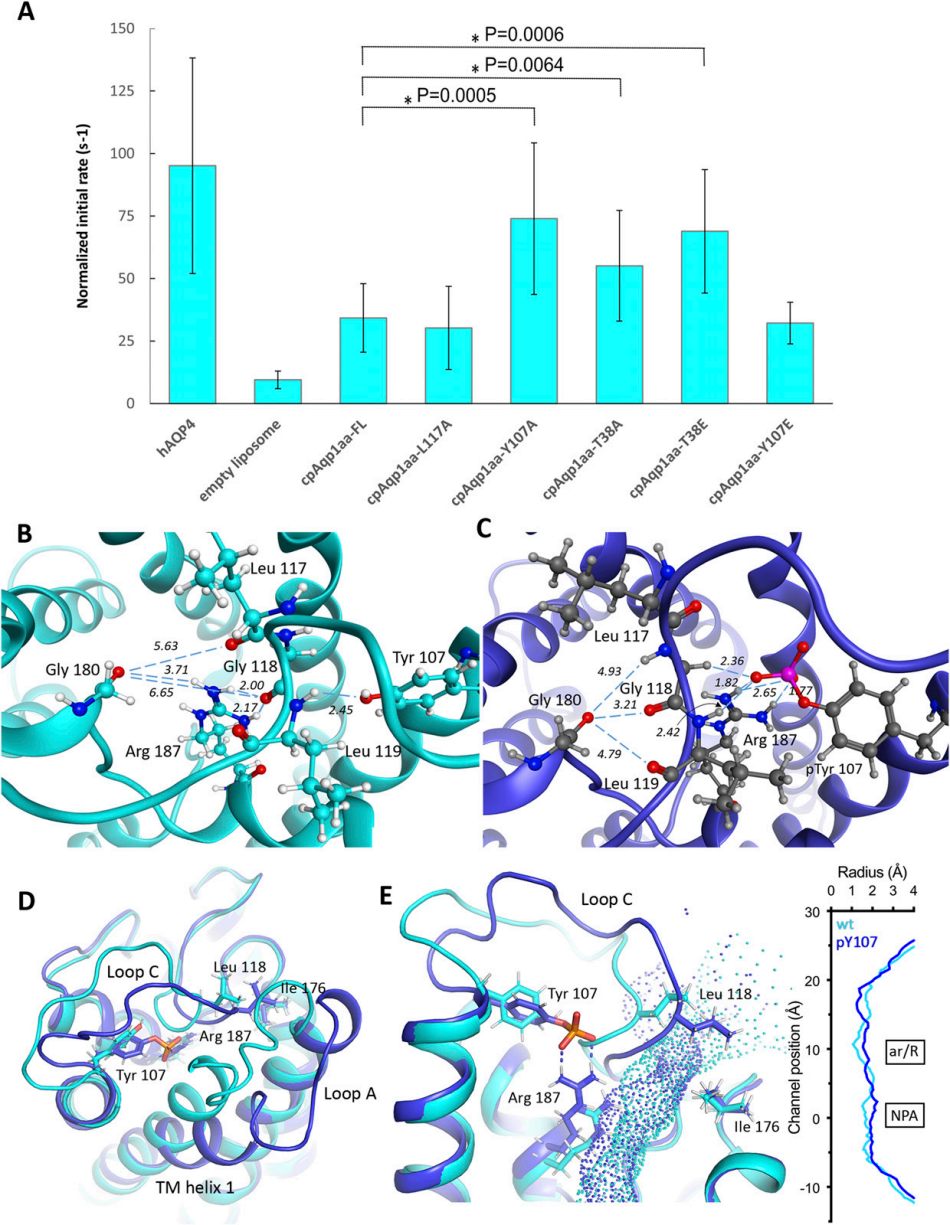
Mutational and in silico analysis of the extracellular constriction region. **(A)** The initial water permeability rates for wild-type and mutant cpAQP1aa with the rate of hAQP4 shown for comparison. All values are normalized taking the protein amount into account (n = 6–31 ± SD). The normalized initial water flow rates show that the Y107A, T38A, and T38E mutants are more efficient than the wild-type protein, in the range of hAQP4 (no significant differences), whereas the L117A and Y107E are similar to wild-type. **(B)** Zoom-in the pore opening in an energy-minimized final snapshot from the 100-ns molecular dynamic simulation of the wild-type system with Tyr 107, Leu 117, Gly 118, Leu 119, Gly 180, and Arg 187 highlighted. Distances are given in Ångström. **(B, C)** Same as (B) but for the pY107 system. The extracellular loop C has shifted to the left, narrowing the pore opening, and is held in place by the interactions between Arg 187 and phosphorylated Tyr 107. **(D, E)** Overlay of the final snapshots from the simulations for wild-type (cyan) and pY107 (blue). In pY107, Arg 187 is pulled away from the pore center because of its interaction with the phosphorylated tyrosine side chain, with its new position causing the distal end of transmembrane helix 1 and loop A to be displaced. **(E)** Analysis of the pore dimensions using HOLE (E) shows that the constriction region (channel position ∼15 Å) is narrower in pY107 than in the wild-type system. In addition, the new position of Arg 187 results in a widening of the ar/R region in pY107.

The structure of cpAQP1aa reveals that loop C forms a unique conformation that restricts the pore at the extracellular side and is stabilized by a hydrogen-bonding network located near Leu 117, specifically involving residues Tyr 107, residues 114–119, and Arg 187 (Fig 2C and D). To evaluate the importance of Leu 117 and Tyr 107 for cpAQP1aa water flow, we mutated these residues to alanine and studied their water permeability in liposomes. As seen in Fig 4A and Table S2, proteoliposomes containing L117A had a similar water flow rate as wild-type cpAQP1aa (normalized values), suggesting that it is not the side chain of Leu 117 in itself that is critical for the restricted water flow of cpAQP1aa but rather the overall fold of the loop C backbone. This is in agreement with the structural analysis, which showed that the narrowest point of the channel is defined by the backbone carbonyls of Leu 117 and Gly 180 and not the Leu 117 side chain (Fig 2C). In contrast, Y107A showed a significantly higher water flow rate (normalized values), in the range of that of hAQP4, suggesting this mutation gives rise to a fully open pore (Fig 4A and Table S2). This strongly supports that the tyrosine side chain is important for forming the network that stabilizes the unique fold of loop C seen in cpAQP1aa.

Table S2 Water flow for cpAQP1aa wild type and mutants.

To identify mutations that may be of further relevance for controlling cpAQP1aa water flow, we analyzed the cpAQP1aa sequence putative phosphorylation sites using the GPS5.0 server (Wang et al, 2020). Two sites of potential interest were identified: Tyr 107 and Thr 38, the latter which is located in loop A, in close vicinity to the loop C constriction region (Fig 2F). We hypothesized that phosphorylation of these two sites could trigger conformational changes of loop C that affect the pore dimensions. To explore this, we created alanine and glutamate mutants of both sites, a common strategy to abolish and mimic phosphorylation, respectively, and assayed their water permeability (Fig 4A, Table S2, and Fig S6C and D). As described above, the Y107A displayed increased water permeability. This was most likely because of the loss of the hydrogen bond between Tyr 107 and loop C. For Y107E, however, the water permeability was similar to wild-type cpAQP1aa. This is consistent with glutamate generally being considered as a poor mimetic of a phosphorylated tyrosine, as opposed to phosphorylated threonine and serine (Anthis et al, 2009). In contrast, both T38A and T38E were significantly more efficient than wild-type (but not significantly different from each other), facilitating water passage at a similar rate as hAQP4 and Y107A. This suggests that mutation of this specific residue could lead to conformational changes that result in altered pore dimensions.

**Figure S7.**
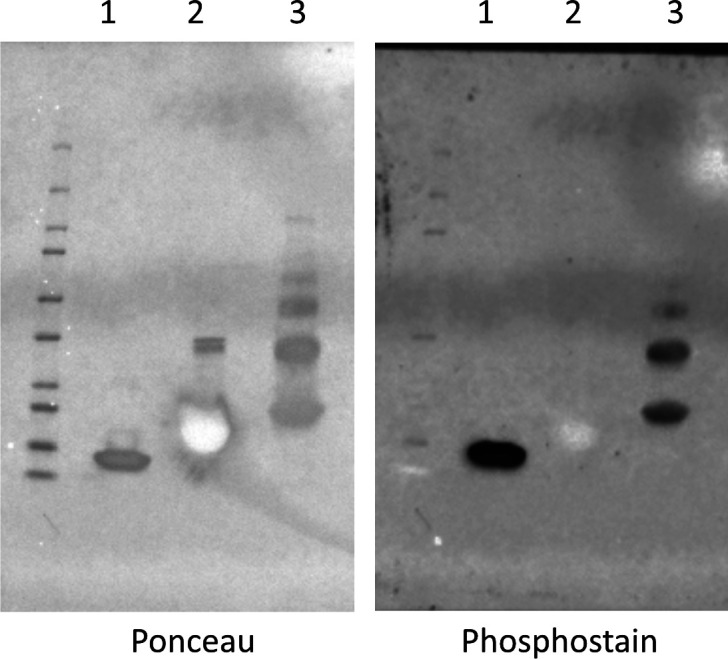
Confirmation of phosphorylation of cpAQP1aa expressed in *Pichia pastoris*. SDS–PAGE and phosphostain for (1) positive control (lysozyme), (2) negative control (SPY), and (3) cpAQP1aa-243.

To rule out that our results were not influenced by cpAQP1aa being phosphorylated by the expression host, as shown previously for AQPs expressed in *P. pastoris* (Ampah-Korsah et al, 2016; Roche et al, 2017), we investigated the phosphorylation status of recombinant cpAQP1aa. First, the protein construct used for crystallization (cpAQP1aa-243) was analyzed by immunoblot using a phosphate-binding tag to identify phosphorylated proteins. The blot showed that cpAQP1aa-243 is indeed phosphorylated (Fig S7). Although the electron density map clearly shows that Tyr 107 is not phosphorylated in the crystal structure (Fig 2D), the phosphorylation status of Thr 38 is more difficult to evaluate because the electron density for this residue is much less clear. Moreover, it cannot be fully ruled out that a fraction of the protein in solution is phosphorylated on either residue but not included in the crystal. We therefore further analyzed the phosphorylation status of full-length cpAQP1aa using mass spectrometry. Phosphorylation was confirmed at three C-terminal residues, Ser 239, Thr 254, and Thr 255 (Fig S1), but despite multiple efforts, no phosphorylation was found on either Thr 38 or Tyr 107. This shows that neither of the constructs used for structural and functional studies are phosphorylated at the two sites proposed to influence the conformation of loop C (Thr 38 or Tyr 107) but may be phosphorylated at sites within the C-terminus that are not visible in the structure because of disorder (Ser 239) or truncation (Thr 234 and 255). As such, we are confident that the functional characterization of wild-type cpAQP1aa rather than their respective alanine mutation gives the most accurate estimate the water transport rate through cpAQP1aa that is not phosphorylated on Tyr 107 or Thr 38.

### MD simulations of loop C

**Figure S8.**
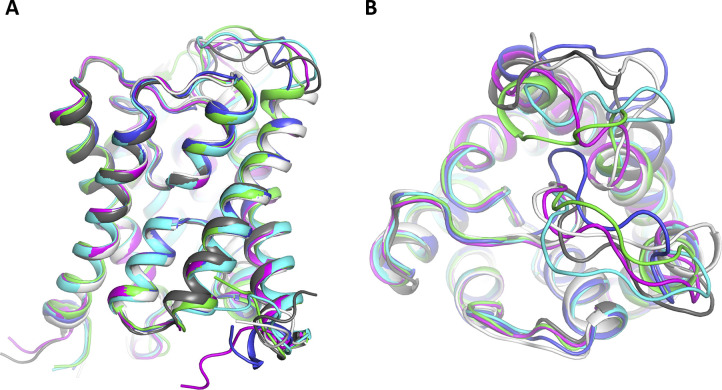
Snapshots from molecular dynamic simulations of wild-type and mutant cpAQP1aa. Overlay of the energy-minimized structures of the final snapshots after 100-ns simulation of wild-type cpAQP1aa (cyan), pY107 (blue), pT38 (magenta), Y107E (green), Y107A (white), and L118A (gray). **(A, B)** Side view and (B) view from the extracellular side. The structures are highly similar except for structural variability in loops A and C.

To shed additional light on the influence of mutations and putative phosphorylation on the conformation of loop C in cpAQP1aa, MD simulations were applied to explore the roles of Thr 38, Tyr 107, and Leu 117. For this purpose, the phosphorylated variants pT38 and pY107 as well as the L117A, Y107A, and Y107E mutants were generated. Noteworthy, all simulated structures are highly stable in the time frame of the current simulations (100 ns) (Fig S8). Similar to the crystal structure (Fig 2D), the MD simulations show a clear role for Tyr 107 and Arg 187 in stabilizing the conformation of loop C that is responsible for forming the extracellular constriction region in cpAQP1aa. Specifically, in the simulated wild-type cpAQP1aa structure, hydrogen bonding is established between the phenolic oxygen of Tyr 107 and the backbone NH of Gly 118 (2.45 Å in the minimized structure after 100-ns MD simulation), and two hydrogen bonds are formed between Arg 187 and the carbonyl oxygen of Gly 118 (distances 2.00 and 2.17 Å) (Fig 4B). In addition, a stable interaction between Arg 198 and the carbonyl of Gly 180 is formed (O - - HN distance 3.71 Å). The distances between the backbone carbonyl oxygen of Gly 180 and those of Leu 117, Gly 118, and Leu 119, representing the pore opening, remain very stable throughout the simulation (5.63, 6.65, and 6.82 Å in the minimized final snapshot, Fig 4B). These values are highly similar values to those obtained for pT38, L117A, Y107A, and Y107E, suggesting that they are in a similar structural state. In contrast, for the pY107 variant, the system gradually adjusts to the bulkiness, altered electrostatics, and hydrogen-bonding conditions of the phosphorylated side chain. During the initial stages of the simulation, Gly 118 is pushed toward Gly 180 by the bulky phosphate group of pY107 (Fig 4C). In addition, the backbone of Leu 117 rotates so that its carbonyl points away from the pore opening and the amine group of Gly 118 instead orients toward Gly 180. After about 50 ns of the production run, there is a rotation of the phosphate group which in turn is accompanied by a motion of Arg 187 from its position near the pore opening, to instead forming two strong hydrogen bonds to the phosphate of pY107. The phosphate group also forms strong interactions to the backbone NH of Leu 119 and a hydrogen of Gly 118. Because of the shift in the loop C orientation, kept in place by the strong interactions to the pY107 – R187 pair, the distance between the carbonyl oxygen of Gly 180 and Gly 118 is shortened to 3.21 Å (and 4.8–4.9 Å to the CO of Leu 119 and NH of Leu 117, respectively). These distances are significantly shorter than the 5.6–6.9 Å observed in the wild-type system. Moreover, to accommodate for the change in the position of Arg 187, the distal part of transmembrane helix 1 is displaced ∼ 6 Å compared with the simulated wild-type system, causing loop A to move away (Fig 4D).

To explore the effect of phosphorylation on the pore dimensions, we analyzed the coordinates from the final stage of the wild-type and pY107 simulations using HOLE (Fig 4E). For wild-type cpAQP1aa, the constriction region is somewhat wider than in the crystal structures. This suggests that there is some room for flexibility in this region and that wild-type cpAQP1aa may be somewhat less restricted in solution than in the crystal structure. In contrast, the shift in the position of loop C in pY107 causes the pore to narrow to a radius of ∼1.2 Å, a similar value as that seen in the crystal structure (Fig 2C). In addition, the pore widens at the ar/R region because of the arginine being pulled away from the pore center by its interaction with the Tyr 107 phosphate group, adopting a pore diameter more similar to aquaglyceroporins. These structural changes around the constriction and ar/R regions suggest that phosphorylation of Tyr 107 could lead to altered pore dimensions and/or solute permeability.

## Discussion

The current understanding of the physiological relevance of AQPs in fish is mainly based on gene identification and phylogenetic analysis and has suggested that fish AQPs are involved in many physiological functions including osmoregulation and passage of gas such as ammonia and CO_2_ (Horng et al, 2015; Talbot et al, 2015). However, the molecular details of fish AQP function and regulation, and hence their role in fish physiology, have been lacking. In the current study, we present the first structural and biochemical analysis of a fish AQP, cpAQP1aa, from the climbing perch, an AQP that has been previously proposed to have its main role in ammonia excretion (Ip et al, 2013). Our studies show that cpAQP1aa is a water-specific AQP that is not permeable to glycerol (Fig S5), comprises a residue composition at the ar/R region that is consistent with water specificity (Fig 2), and lacks the structural features that are believed to be associated with ammonia passage (Fig 3). Given its high expression in gills and skin, tissues that are in direct contact with the fish surroundings (Ip et al, 2013), it therefore seems plausible that cpAQP1aa is involved in osmoregulation.

The most striking feature of the cpAQP1aa structure is the presence of a constriction region at the extracellular side of the pore formed by a unique conformation of loop C (Fig 2C and D). From a sequence comparison, it is not obvious why the conformation of loop C differs in cpAQP1aa compared with other structurally characterized AQPs (Fig S1). A possible explanation lies in the hydrogen-bonding network surrounding Leu 117 which, through interactions with Tyr 107, stabilizes loop C in its unique conformation (Fig 2D). Interestingly, Tyr 107 is rather well-conserved among fish species and present in several mammalian AQP homologs, including AQP0, AQP4, and AQP7 (Fig S1), However, in mammalian AQPs, the tyrosine side chain adopts a different position, pointing away from the pore rather than toward it (Fig 2F). Furthermore, in cpAQP1aa, Tyr 107 is followed by a glycine, a residue that is highly conserved in fish but varies in other eukaryotic AQPs (Fig S1). It may be that the combination of tyrosine and glycine at these positions is necessary for the unique conformation of loop C seen in cpAQP1aa. Furthermore, the conservation of the Tyr–Gly motif in fish suggests that the extracellular constriction region observed in cpAQP1aa may be a general feature among fish AQPs.

**Figure S9.**
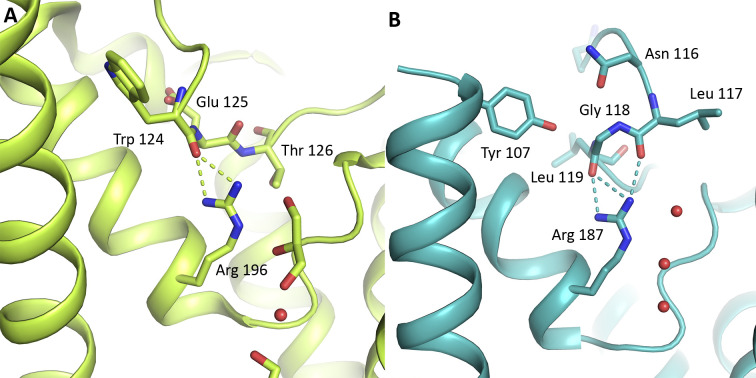
Comparison of PfAQP and cpAQP1aa. **(A)** Crystal structure of *Plasmodium falciparum* AQP (PfAQP, PDB code 3C02) showing the interaction between Arg 196 of the ar/R.-region and the main chain carbonyl of Trp 124 in loop C. **(B)** Similar interaction between Arg 187 and the main chain of loop C is seen in cpAQP1aa. The resulting saturation of the arginine guanidino group hydrogen-bonding sites has been suggested to play a role in water selectivity. Water and glycerol molecules in the PfAQP and cpAQP1aa pores are shown in sphere and stick representation, respectively.

In addition to restricting the diameter of the pore, the conformation of loop C also allows for the formation of hydrogen bonds between Arg 187 of the ar/R region and the loop C backbone, specifically the carbonyls of Leu 117 and Gly 118. A similar interaction between the arginine and loop C was also seen in the crystal structure of the AQP from the malaria parasite *Plasmodium falciparum* (PfAQP) (Fig S9) (Newby et al, 2008). Through this interaction, the hydrogen-bonding sites of the arginine guanidino group are saturated, a feature that is typically seen in water-specific AQPs but not aquaglyceroporins. Based on mutational and structural analysis of PfAQP, it was proposed that the hydrogen-bonding pattern of the arginine in combination with a wide ar/R region underlies its unusual ability of being permeable to both glycerol and water at a high rate (Beitz et al, 2004; Newby et al, 2008). Given that the conformation of loop C allows similar interactions to occur also in cpAQP1aa, it may be that it also plays a role in solute selectivity and the permeability rate.

**Figure 5.**
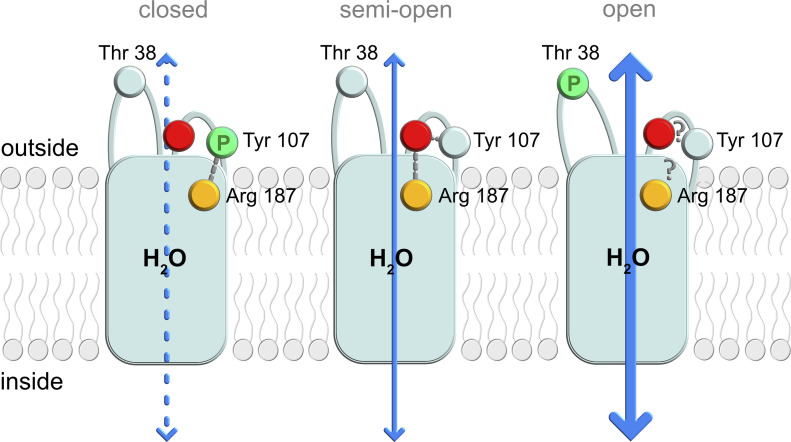
Proposed regulatory mechanism for cpAQP1aa. Schematic of a possible extracellular gating mechanism where phosphorylation of Thr 38 and Tyr 107 controls the water permeability. The crystal structure represents the non-phosphorylated state (middle) in which a hydrogen-bonding network between Tyr 107, backbone residues in the distal part of loop C (represented by a red sphere), and the ar/R region arginine (Arg 187, orange sphere) results in an extracellular constriction region that is consistent with a gate caught in a semi-open state. Upon phosphorylation of Tyr 107 (left), this hydrogen-bonding network is rearranged, and the phosphorylated tyrosine side chain instead interacts with Arg 187. This causes structural changes in this region, including further narrowing of the constriction region that is indicative of pore closure and a widened ar/R region. Phosphorylation of Thr 38 (right) results in an open pore; however, because of the structural flexibility of this region, a possible structural mechanism for this remains elusive.

Although the conformation of the cpAQP1aa loop C does not cause complete pore closure, it is possible that a trigger such as phosphorylation or pH could cause structural changes of the loop that modulate water flow, as seen for SoPIP2;1 (Tornroth-Horsefield et al, 2006). In this respect, it is interesting to note the presence of two putative phosphorylation sites, Tyr 107 and Thr 38, within this region (Fig 2F). We hypothesize that the crystal structure of cpAQP1aa is caught in a semi-open state and that, similarly as for SoPIP2;1, the conformation of loop C could be subject to phosphorylation-induced changes that result in gating from the extracellular side (Fig 5). Although gating of cpAQP1aa remains to be shown in vivo, there are several key findings presented here that support this hypothesis. The water permeability of cpAQP1aa is similar to that of the gated PIP2;1 from spinach (SoPIP2;1), which has been shown to only reach its full capacity when locked in an open state by a phospho-mimicking mutation (Nyblom et al, 2009). This is also the case for cpAQP1aa, for which mutation of a putative phosphorylation site at Thr 38 in extracellular loop A increases the water permeability of cpAQP1aa to a similar level as that of hAQP4 (Fig 4A). Thr 38 is not a conserved residue among fish species (Fig S1) but appears in the frog, which opens up for speculations on a general osmoregulatory mechanism for an organism living both on land and in water. In the crystal structure, Thr 38 is located in a highly flexible region with less well-defined electron density, wherefore its exact position and conformation is uncertain. This may explain why MD simulations failed to capture any phosphorylation-dependent structural changes within the simulated timescale. Hence, a structural mechanism for how phosphorylation of Thr 38 may result in an open pore remains elusive.

In contrast to Thr 38, the second putative phosphorylation site in this region, Tyr 107, is highly conserved in fish (Fig S1), and its key role in stabilizing loop C in its unique position is clear (Figs 2D and 4A). Moreover, the MD simulations suggests significant structural perturbations when this residue is phosphorylated (Fig 4D). Although phosphorylation of Tyr 107 did not result in a fully closed pore, the observed structural changes results in pore narrowing as compared with the wild-type system (Fig 4E and F). It may be that this represents the initial structural events that follow Tyr 107 phosphorylation and that the transition to a fully open pore is not caught within the time frame of the simulations. The additional observation of a widened ar/R region consistent with an aquaglyceroporin is intriguing and indicates a possible effect also on solute selectivity. However, because of the narrowness of the constriction region, further structural changes would be needed to allow glycerol to permeate the pore.

**Figure S10.**
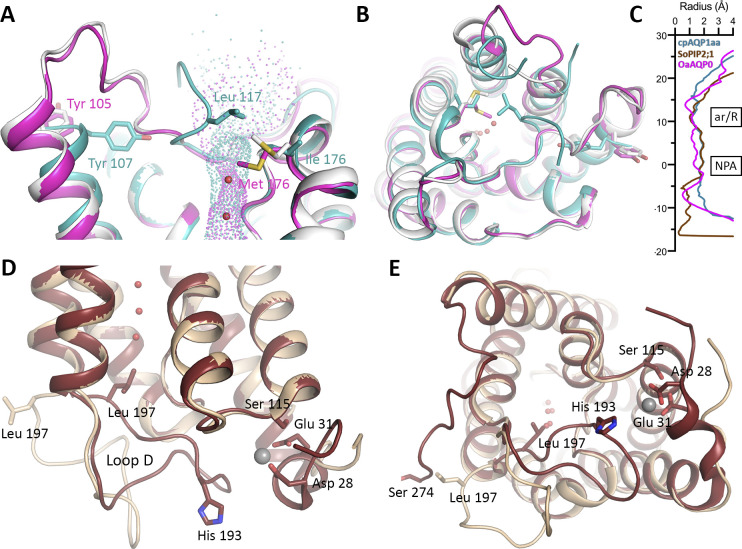
Structural comparison with other gated aquaporins. **(A, B)** Overlay of cpAQP1aa (teal) and mammalian AQP0 in its junctional (magenta, PDB code 2B6O) and non-junctional (white, PDB code 1YMG) form viewed (A) parallel to the membrane and (B) from the extracellular side. **(A)** Pore profiles of junctional AQP0 and cpAQP1aa as calculated by HOLE are indicated by small spheres in (A). In junctional (closed, magenta) AQP0, the side chain of Met 176 points into the channel, causing restriction, whereas in non-junctional AQP0 (open, white), it points away from the channel. In cpAQP1aa, this position corresponds to Ile 176 which together with Leu 117 and Gly 180 constitutes an extracellular constriction site. The cpAQP1aa constriction site is a result of a unique conformation of loop C, whereby the side chain of Tyr 107 pushes it toward the center of the channel. In AQP0, the corresponding residue Tyr 105 points in the opposite direction, as seen for other AQPs. **(C)** Comparison of the pore profiles of cpAQP1aa (teal), junctional AQP0 (magenta), and spinach PIP2;1 (SoPIP2;1, brown) as calculated by HOLE. Constriction regions at the extracellular side of the pore are observed in cpAQP1aa and AQP0, whereas cytoplasmic constriction regions are present in AQP0 and SoPIP2;1. **(D, E)** Crystal structures of SoPIP2;1 in closed (brown, PDB code 1Z98) and open (beige, PDB code 2B5F) conformations viewed (D) parallel to the membrane and (E) from the cytoplasmic side. Channel closure is achieved through a conformational change of loop D, resulting in the insertion of Leu 197 into the channel opening. A Ca^2+^-binding site at the N-terminus, occupied by Cd^2+^ in the crystal structure that is involved in stabilizing loop D in the closed conformation is indicated by a gray sphere. Residues involved in gating by pH (His 193), phosphorylation (Ser 115 and Ser 274), and the Ca^2+^-ligands (Asp 28 and Glu 31) are highlighted in stick representation.

Extracellular gating has previously been proposed for mammalian AQP0 found in junctions in the eye lens fiber cells, for which a pinching-type mechanism was proposed. This was based on the observation that the Met 176 side chain protrudes into the channel in the junctional (closed) form (Gonen et al, 2005) (Fig S10A and B). As seen in Fig S1, Met 176 in AQP0 is a unique residue at this position among the AQPs; most isoforms have isoleucine at this site. In cpAQP1aa, this residue corresponds to Ile 176, which together with Leu 117 and Gly 180 forms the putative extracellular gate (Fig S10B and C). However, in comparison to mammalian AQP0, the proposed cpAQP1aa gating mechanism is more consistent with capping through a conformational change of loop C. As such, gating of cpAQP1aa would be more similar to that of SoPIP2;1 (Tornroth-Horsefield et al, 2006) for which a conformational change of a loop brings a leucine residue closer to the center of the pore end, albeit at the opposite side of the membrane (Figs 2 and S10C–E).

**Figure S11.**
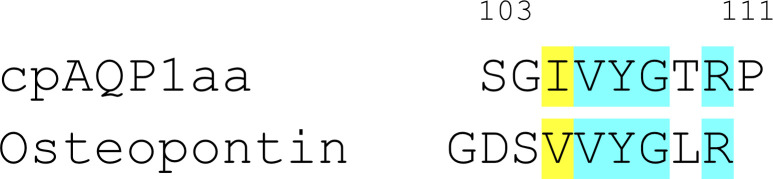
Putative phosphorylation site in cpAQP1. Extracellular tyrosine kinase PKDCC, also known as vertebrate lonesome kinase, has been shown to phosphorylate the protein osteopontin that has a very similar sequence as cpAQP1aa at Tyr 107. This supports the possibility that cpAQP1aa is phosphorylated in vivo, even though this kinase is suggested to not exist in yeast.

The possible involvement of extracellular phosphorylation sites in the proposed cpAQP1aa gating mechanism is intriguing. All other known phosphorylation-dependent AQP gating mechanisms involve cytoplasmic phosphorylation sites (Nesverova & Tornroth-Horsefield, 2019), and to our knowledge, this is the first time extracellular phosphorylation has been proposed to control gating of any membrane channel. Nevertheless, extracellular phosphorylation is well established, and several extracellular kinases have been characterized in mammals (Kinoshita et al, 2009; Bordoli et al, 2014; Cui et al, 2015; Tagliabracci et al, 2015). For example, the secreted tyrosine kinase vertebrate lonesome kinase (VLK) has been shown to influence the localization and regulation of a synaptic receptor, supporting the role of extracellular phosphorylation in controlling membrane protein function (Hanamura et al, 2017). These extracellular kinases, including VLK, are also present in *A. testudineus*. Furthermore, VLK has been shown to be able to phosphorylate sequences that are highly similar to the sequence around Tyr 107 (Fig S11) (Bordoli et al, 2014), supporting this as a likely kinase candidate. In the case of Thr 38, it is more difficult to identify the most likely candidate out of several secreted Ser/Thr kinases, but a member of the recently identified secreted kinase family FAM69 (DIA1) is predicted to phosphorylate the same sequence (Ser Thr Pro) and is one possibility (Dudkiewicz et al, 2013; Hareza et al, 2018). The fact that neither VLK nor DIA1 is found in yeast could explain why cpAQP1aa was not phosphorylated at these sites by the expression host.

To conclude, the special characteristic of the climbing perch is its ability to both be truly euryhaline and be able to spend time on land and in air. Under these conditions, it is likely that the presence of gated AQPs is beneficial in order for the fish to finely regulate the water flow across its epithelia. Our structural and functional characterization of an aquaporin from this unique fish species has given novel insights into fish water homeostasis, including possible adaptation mechanisms that allow fish to withstand the osmolarity changes that arise from these large environmental variations.

## Materials and Methods

### Cloning and overproduction

The full-length gene for *A. testudineus*, climbing perch, aquaporin 1aa (cpAQP1aa-FL, residues 1–261) (Genbank accession code JX645188.1) was codon-optimized for protein production in *P. pastoris* (Genscript), including GCT after the first methionine to meet the yeast consensus sequence (Romanos et al, 1992) and sub-cloned into the pPICZA vector (Invitrogen), fused with a C-terminal 8xHis-tag (cpAQP1aa-FL). Two truncated constructs, untagged-cpAQP1aa-243 (residues 1–243) and C-terminal 8×His-tagged-cpAQP1aa-243, were prepared for crystallization. The genes were ligated into the pPICZA vector between the *EcoR*I and *Xho*I or *Not*I restriction sites, respectively. The plasmids were linearized by the restriction enzyme *Mss*I (Thermo Fisher Scientific) and transformed into an aquaporin-deficient Δ33 strain of *P. pastoris* by electroporation (Fischer et al, 2009). For all the constructs of cpAQP1aa used in this study, extra nucleotides “GCT” encoding an alanine were intentionally added after the start codon “ATG” to meet the Kozak consensus sequence (Öberg et al, 2011a), and the first methionine is numbered as Met0.

Yeast clone selection was performed on YPD plates containing 2,000 μg/ml Zeocin (Öberg et al, 2011a), and small-scale protein production was tested for selected strains. Membrane fractions were analyzed by SDS–PAGE and immunoblot. Strains with the highest production of His-tagged proteins were picked for large-scale production in 3-liter fermentors. For untagged-cpAQP1aa-243, the strain corresponding to the largest colony on the high-Zeocin plate was used for fermentation directly. Protein production was induced by methanol fed-batch, typically lasting between 24 and 48 h, resulting in a yield of more than 250 g wet cells per liter culture. Cells were harvested by centrifugation (6,000*g*, 45 min, 4°C) and stored at −20°C.

### Membrane preparation, solubilization, and protein purification

For cpAQP1aa-FL, 60 g of cells were thawed at room temperature and resuspended in 200 ml breaking buffer (50 mM KH_2_PO_4_ [pH 7.5] and 5% [wt/vol] glycerol), supplemented with Complete EDTA-free protease inhibitor (Roche). A Bead Beater (BioSpec) was used to break the cells with 0.5-mm glass beads (Scientific Industries) grinding for 12 × 30 s, with 30-s cooling between runs. Cell debris was separated by a first centrifugation (10,000*g*, 30 min, 4°C) followed by a second centrifugation (15,000*g*, 30 min, 4°C). The crude membrane was harvested from the supernatant by ultracentrifugation (100,000*g*, 90 min, 4°C). The resultant membrane was washed with urea buffer (4 M urea, 5 mM Tris–HCl [pH 9.5], 2 mM EDTA, and 2 mM EGTA) and ultracentrifuged again at 158,000*g* for 90 min at 4°C. A final wash was performed to remove traces of urea by homogenizing the membrane in membrane resuspension buffer (20 mM Tris–HCl [pH 8.3], 300 mM NaCl, 10% glycerol, and 2 mM β-mercaptoethanol [β-MeOH]) and centrifuging at 158,000*g* for 1 h at 4°C. Finally, the membrane was homogenized in the membrane resuspension buffer and stored at −80°C. For untagged-cpAQP1aa-243 and His-tagged-cpAQP1aa-243, the same procedure was used except that the pH of the membrane resuspension buffer was adjusted to eight.

Membrane solubilization of cpAQP1aa-FL was carried out by mixing the membrane with solubilization buffer (20 mM Tris–HCl [pH 8.3], 300 mM NaCl, 10% glycerol, 4% [wt/vol] n-nonyl-β-D-glucopyranoside [NG, Anatrace], and 2 mM β-MeOH) in a 1:1 volume ratio supplemented with Complete EDTA-free protease inhibitor (Roche). The mixture was gently rocked for 1 h in a cold room. Unsolubilized material was removed by ultracentrifugation (149,000*g*, 30 min, 4°C), and the supernatant was transferred to a falcon tube containing pre-equilibrated Ni-NTA agarose beads (QIAGEN) and rocked for 3 h or overnight. 5 mM imidazole was added 20 min before centrifugation to avoid nonspecific binding. The supernatant containing unbound proteins was discarded after centrifugation at 3,000*g* for 3 min at 4°C. Nonspecifically bound proteins were removed by washing the matrix in 15 ml wash buffer (20 mM Tris–HCl [pH 8.3], 300 mM NaCl, 10% glycerol, 0.4% NG, 50 mM imidazole, and 2 mM β-MeOH) for three times, with centrifugation between washes (3,000*g*, 3 min, 4°C). Thereafter, the resin with bound protein was transferred to a 6-ml gravity-flow column (QIAGEN). Protein was eluted with 10 ml elution buffer (20 mM Tris–HCl [pH 8.3], 300 mM NaCl, 10% glycerol, 0.4% NG, 350 mM imidazole, and 2 mM β-MeOH). Protein fractions were analyzed using SDS–PAGE. Protein-containing fractions were pooled together, and buffer was exchanged to gel filtration buffer (20 mM Tris–HCl [pH 8.3], 100 mM NaCl, 10% glycerol, 0.4% NG, and 2 mM β-MeOH) immediately using a PD-10 column (GE Healthcare). The protein eluted from the column was concentrated to 500 μl using a 50-kD cut-off concentrator (Merck Millipore), filtered through a 0.2-μm centrifugal filter (VWR), and injected to a Superdex 200 increase gel filtration column (GE Healthcare), pre-equilibrated with gel filtration buffer. Fractions were analyzed using SDS–PAGE, and the protein-containing fractions were pooled together. For crystallization, the protein was concentrated to ∼18 mg/ml and stored at −80°C. For His-tagged-cpAQP1aa-243, the protocols were same except that the pH of all the buffers was eight.

For the untagged protein, the solubilization buffer contained 20 mM Tris–HCl (pH 8), 10% glycerol, 4% NG, and 2 mM β-MeOH. After solubilization and centrifugation, the solubilized material was diluted in a 1:3 (vol/vol) ratio with dropwise addition of pre-chilled dilution buffer (20 mM MES [pH 6], 10% glycerol, 0.4% NG, and 2 mM β-MeOH). The diluted sample was filtered using a 0.2-μm bottle top filter (Thermo Fisher Scientific) and applied to a Resource S cation exchange column (GE Healthcare) pre-equilibrated with buffer A (20 mM MES [pH 6], 37.5 mM NaCl, 10% glycerol, 0.4% NG, and 2 mM β-MeOH) at a flow rate of 0.5 ml/min. After washing the column with 10 column volume of buffer A, protein was eluted with the gradient 0–40% of buffer B (20 mM MES [pH 6], 1 M NaCl, 10% glycerol, 0.4% NG, and 2 mM β-MeOH). Fractions were analyzed using SDS–PAGE, and the protein-containing fractions were pooled together and concentrated to 500 μl and injected to a Superdex 200 increase column (GE Healthcare) pre-equilibrated with gel filtration buffer (20 mM Tris–HCl [pH 8], 100 mM NaCl, 10% glycerol, 0.4% NG, and 2 mM β-MeOH). The untagged protein was analyzed, concentrated, and saved as described above. The identity of all proteins was verified by mass spectrometry.

### Optimization of the production of *cpAQP1* in *P. pastoris*

The full-length cpAQP1aa was successfully produced to high yields in the *P. pastoris* membranes, with an estimated yield of 63 mg highly pure protein per liter culture after a two-step purification procedure. This protein was used for functional characterization after reconstitution into liposome using stopped flow. Crystallization attempts were also pursued, but no crystals were achieved. In the optimization of the crystallization approach for cpAQP1aa, truncated versions of the protein were evaluated, where truncations were based on the alignment with vertebrate AQPs with available structures. The N-terminus of cpAQPaa is very short, and the focus for truncations leaned on the C-terminus of the protein which specifically aligned with the C-terminal domain of mammalian AQP0, 1, 2, and 5 (see Fig S1). Based on this alignment, six different C-terminal truncations were made from cpAQP1aa residue 228–243, where the shortest truncation showed the highest production level (38 mg/l), and it was therefore taken further to large-scale production and crystallization. Resolution down to 7 Å was achieved for this truncated version of cpAQP1aa, and based on inhomogeneity and instability during purification, three additional versions of the same construct were evaluated including an untagged variant because it was suspected that the His-tag had a negative influence on the crystallization event. This hypothesis turned out to be valid; a monodisperse peak appeared after size-exclusion chromatography, indicating a homogenous population of stable protein. Importantly, highly ordered crystals were achieved for this version, and a diffraction at 1.9 Å was achieved (Table S1).

### Phosphostain

The aquaporin from the climbing perch, cpAQP1aa-243, was analyzed for phosphorylation together with a positive and negative control, lysozyme, and the periplasmic chaperone spy, respectively, by using a selective phosphate-binding tag molecule (BTL-111), with streptavidin-conjugated HRP bound to it (prepared according the manual from Fujifilm Wako Pure Chemical Corporation). An SDS–PAGE is performed by loading 7.5 μg of each protein species and operated at 300 V for 15 min. After transferring the proteins to a PVDF membrane, the instruction manual is followed, which briefly consists of a soaking step with 1 × TBS-T for at least 1 h, 30 min of incubation with the tag molecule and consecutive washing steps. The blot was developed by using 4 ml of Genescript LumiSensor chemiluminescent HRP substrate.

### X-ray crystallography

Crystallization trials were carried out for all the constructs, and the best crystals were obtained for untagged-cpAQP1aa-243 by the hanging-drop vapor diffusion method at 4°C. The reservoir solution contained 100 mM Tris–HCl (pH 7.8), 5% poly-γ-glutamic acid low–molecular-weight polymer (γ-PGA, Na+ form, LM) and 30% (vol/vol) PEG400. Before setting up the crystallization drops, 4 μl of the reservoir solution was mixed with 1 μl 30% (wt/vol) D-sorbitol. For the crystals at lower pH, the reservoir solution contained 300 mM lithium sulfate, 100 mM ADA (pH 6.5), and 30% (vol/vol) PEG400; and 4 μl of the reservoir solution was mixed with 1 μl 30% (wt/vol) dextran sulfate sodium salt (Mr 5,000). Crystallization drops were then set up by mixing the reservoir/additive mixture with protein at 1:1 or 2:1 ratio, and the drops were left to equilibrate against 0.5 ml reservoir at room temperature, and crystals grew in cold room (4°C). Crystals with different shapes appeared after a few days. Crystals were mounted in cryo-loops, flash frozen in liquid nitrogen, and stored for data collection.

### Data collection and structural determination

Complete data sets were collected at beam line P13 (wavelength 0.8 Å) at the PETRA III storage ring (DESY) (Cianci et al, 2017) and at beam line ID30B (wavelength 0.911647 Å) at European Synchrotron Radiation Facility (Svensson et al, 2015), France, for the low pH crystals. Data sets were processed using MOSFLM (Leslie, 1992) and scaled using SCALA of the CCP4 program suite (Collaborative Computational Project Number 4, 1994). Molecular replacement was carried out using the program Phaser from the CCP4 program suite (Collaborative Computational Project Number 4, 1994). A homology model of cpAQP1aa based on the crystal structure of bovine AQP1 (PDB: 1J4N) (Sui et al, 2001) was generated using the SWISS-MODEL web server (Biasini et al, 2014). The model was subjected to iterative rounds of model building and refinement in COOT (Emsley & Cowtan, 2004) and Phenix (Afonine et al, 2012). The final models consists of residues 1–34, 38–108, 114–226, and 124 water molecules for the structure at pH 7.8 and residues A2-34, 38–110, and 112–225; residues B4-34, 38–110, and 112–225; residues C4-226; and residues D1-34, 37–226 for the structure at pH 6.5. All structure figures were made in PyMOL (The PyMOL Molecular Graphics System, Version 2.3.1, Schrödinger, LLC). All MD simulations were conducted using the MD engine (Krieger & Vriend, 2015) in the YASARA program (Krieger & Vriend, 2014), with the AMBER14 force field (Maier et al, 2015).

### Water and glycerol permeability studies

Water permeability studies were carried out using similar protocols described in Öberg et al (2011b) and Isaksson et al (2017). Liposome stock (25 mg/ml) was prepared by homogenizing *Escherichia coli* polar lipid extract (Avanti Polar Lipids) in reconstitution buffer (50 mM NaCl and 50 mM Tris–HCl [pH 8.3] for cpAQP1aa-FL and pH 8.0 for His-tagged-cpAQP1aa-243). An n-octyl-β-D-glucoside (β-OG; Anatrace) stock solution (50 mM Tris–HCl [pH 8.0], 50 mM NaCl, and 10% β-OG [wt/vol]) was added to the liposome solution to a final concentration of 1%. Purified protein was added into the mixture with a theoretical lipid-to-protein ratio of 50. Samples were gently mixed and incubated for 10 min. Subsequently, Biobeads (Bio-Rad Laboratories) was added to the mixture to remove the detergents by incubation (and rocking) at room temperature in dark overnight. The resulting proteoliposome was pelleted (100,000*g*, 1 h, 10°C) and gently resuspended in reconstitution (same as described before) buffer to a final lipid concentration of 2 mg/ml. Samples were filtered using a 0.2-μm spin column filter (VWR) by centrifugation (10,000*g*, 3 min, 20°C) before measurements. Control liposomes were prepared using the same protocol but with addition of corresponding gel filtration buffer instead of purified protein.

Water flow experiments were conducted on an SFM 2000 (BioLogic Science Instruments). In brief, 88 μl of sample was rapidly mixed with the same amount of hyperosmolar solution (reconstitution buffer with 300 mM sucrose). Change in light-scattering intensity at the wavelength of 438 nm was monitored at the fixed angle of 90°. At least two independent proteoliposome preparations were made for each target protein, and three samples were measured for each. For each sample, three measurements were averaged for each curve, providing the raw data for the calculation of the initial rate. The light-scattering data were normalized and fitted to a two-exponential equation:

$$I=A_{1}e^{-k_{1}\left(t-t_{0}\right)}+A_{2}e^{-k_{2}\left(t-t_{0}\right)}+A_{3}$$ (1) ,

where *I* is the intensity corresponding to the time point *t*; *A*_1_, *A*_2_, and *A*_3_ are coefficients; *k*_1_ and *k*_2_ are the rate constants of water flow; and *t*_0_ is the dead time of the measurements.

Averaged curves (n = 6–31 [2–11]), where the number of individual preparations are given in parenthesis, were used to calculate the initial rate. Average of the initial rate, derived from the two-exponential equations, plus SD was the calculated for each target. Figures were made using Graphpad Prism version 6.0, and the error bars represent SD. The amount of protein in the liposomes was estimated using immunoblots, and the intensity of the immunoblot signal was quantified for each protein target (n ≥ 2). From this, a normalization factor relative full-length cpAQP1aa was calculated and used to calculate normalized k-values for all protein targets. The relationship between protein amount in the protein liposome and the initial rate is not necessarily linear, but the correlation gives an indication of the real differences in water flow rate for each mutant. Statistical significance was evaluated using unpaired *t* test, and results shown in the bar graphs as stars together with the two tailed *P*-value for each analysis.

Glycerol flow measurements were performed using the same setup with the difference that the liposomes were preloaded with glycerol buffer (300 mM glycerol, 50 mM Tris [pH 8.3], and 50 mM NaCl), and the flow of glycerol out of the proteoliposome was measured via light scattering at 90° and a wavelength of 438 nm. For each measurement, 88 μl glycerol buffer and 88 μl sucrose buffer (300 mM sucrose, 50 mM Tris [pH 8.3], and 50 mM NaCl) were introduced, assuring that the osmolarity of 528 mOsm was applied. For each experiment, a minimum of three stopped-flow measurement traces were averaged and fitted to a two-exponential function using R^2^ values of at least 0.95.

### Molecular dynamics simulations

All MD simulations were conducted using the MD engine (Krieger & Vriend, 2015) in the YASARA program (Krieger & Vriend, 2014), with the AMBER14 force field (Maier et al, 2015). The crystal structure of the aquaporin was first protonated assuming a pH of 7 and energy-minimized. The different mutants or phosphorylated variants (pY107, pT38, Y107A, L117A) were generated by modifying the corresponding residue of the minimized structure in silico, followed by additional energy minimization. Each system was placed in a cubic water box extending 10 Å away from nearest protein atom in each direction, water molecules were randomly replaced by Na^+^ or Cl^−^ ions to ensure charge neutrality, and assuming a physiological salt concentration of 0.9% NaCl.

Following a stepwise sequence of equilibration, the 100-ns production runs were performed on wild-type crystal structure and the five modified proteins, assuming T = 298 K, as maintained through velocity rescaling, and simulation box rescaling to ensure water density of 0.997 for pressure control. Each simulation box contained in the order of 55,000 atoms. All simulations were performed using the default macro md.run in the YASARA program. Analyses were performed with the macro md_analyze in the YASARA program, to which analysis of the bond distances were added.

## Data Availability

The atomic coordinates and structure factors (PDB ID: 7W7S and 7W7R) have been deposited in the Protein Data Bank (http://wwpdb.org/). Structures and trajectories from MD simulations are available at https://zenodo.org/ with DOI: 10.5281/zenodo.7094143.

## Supplementary Material

Reviewer comments

## References

[bib1] Afonine PV, Grosse-Kunstleve RW, Echols N, Headd JJ, Moriarty NW, Mustyakimov M, Terwilliger TC, Urzhumtsev A, Zwart PH, Adams PD (2012) Towards automated crystallographic structure refinement with phenix.refine. Acta Crystallogr D Biol Crystallogr 68: 352–367. 10.1107/s0907444912001308 22505256 PMC3322595

[bib2] Ampah-Korsah H, Anderberg HI, Engfors A, Kirscht A, Norden K, Kjellstrom S, Kjellbom P, Johanson U (2016) The aquaporin splice variant NbXIP1;1α is permeable to boric acid and is phosphorylated in the N-terminal domain. Front Plant Sci 7: 862. 10.3389/fpls.2016.00862 27379142 PMC4909777

[bib3] Anthis NJ, Haling JR, Oxley CL, Memo M, Wegener KL, Lim CJ, Ginsberg MH, Campbell ID (2009) β integrin tyrosine phosphorylation is a conserved mechanism for regulating talin-induced integrin activation. J Biol Chem 284: 36700–36710. 10.1074/jbc.m109.061275 19843520 PMC2794784

[bib4] Beitz E, Pavlovic-Djuranovic S, Yasui M, Agre P, Schultz JE (2004) Molecular dissection of water and glycerol permeability of the aquaglyceroporin from Plasmodium falciparum by mutational analysis. Proc Natl Acad Sci U S A 101: 1153–1158. 10.1073/pnas.0307295101 14734807 PMC337022

[bib5] Biasini M, Bienert S, Waterhouse A, Arnold K, Studer G, Schmidt T, Kiefer F, Cassarino TG, Bertoni M, Bordoli L, (2014) SWISS-MODEL: Modelling protein tertiary and quaternary structure using evolutionary information. Nucleic Acids Res 42: W252–W258. 10.1093/nar/gku340 24782522 PMC4086089

[bib6] Bordoli MR, Yum J, Breitkopf SB, Thon JN, Italiano JE Jr., Xiao J, Worby C, Wong SK, Lin G, Edenius M, (2014) A secreted tyrosine kinase acts in the extracellular environment. Cell 159: 955–1044. 10.1016/j.cell.2014.10.038 PMC4149754 25171405

[bib7] Chang EWY, Loong AM, Wong WP, Chew SF, Wilson JM, Ip YK (2007) Changes in tissue free amino acid contents, branchial Na+/K+ -ATPase activity and bimodal breathing pattern in the freshwater climbing perch, Anabas testudineus (Bloch), during seawater acclimation. J Exp Zool A Ecol Genet Physiol 307: 708–723. 10.1002/jez.a.424 17963240

[bib8] Cianci M, Bourenkov G, Pompidor G, Karpics I, Kallio J, Bento I, Roessle M, Cipriani F, Fiedler S, Schneider TR (2017) P13, the EMBL macromolecular crystallography beamline at the low-emittance PETRA III ring for high- and low-energy phasing with variable beam focusing. J Synchrotron Radiat 24: 323–332. 10.1107/s1600577516016465 28009574 PMC5182027

[bib9] Collaborative Computational Project Number 4 (1994) The CCP4 suite: Programs for protein crystallography. Acta Crystallogr D Biol Crystallogr 50: 760–763. 10.1107/s0907444994003112 15299374

[bib10] Cui J, Xiao J, Tagliabracci VS, Wen J, Rahdar M, Dixon JE (2015) A secretory kinase complex regulates extracellular protein phosphorylation. Elife 4: e06120. 10.7554/elife.06120 25789606 PMC4421793

[bib11] Cutler CP, Cramb G (2000) Water transport and aquaporin expression in fish. In Molecular Biology and Physiology of Water and Solute Transport. Hohmann S, Nielsen S (eds.). pp 431431–441441. London: Kluwer Academic Press.

[bib12] Davenport J, Matin AKMA (1990) Terrestrial locomotion in the climbing perch, anabas-testudineus (bloch) (anabantidea, pisces). J Fish Biol 37: 175–184. 10.1111/j.1095-8649.1990.tb05938.x

[bib13] de Mare SW, Venskutonyte R, Eltschkner S, de Groot BL, Lindkvist-Petersson K (2020) Structural basis for glycerol efflux and selectivity of human aquaporin 7. Structure 28: 215–222.e3. 10.1016/j.str.2019.11.011 31831212

[bib14] Dudkiewicz M, Lenart A, Pawlowski K (2013) A novel predicted calcium-regulated kinase family implicated in neurological disorders. PLoS One 8: e66427. 10.1371/journal.pone.0066427 23840464 PMC3696010

[bib15] Emsley P, Cowtan K (2004) Coot: Model-building tools for molecular graphics. Acta Crystallogr D Biol Crystallogr 60: 2126–2132. 10.1107/s0907444904019158 15572765

[bib16] Evans DH (2008) Teleost fish osmoregulation: What have we learned since August Krogh, Homer Smith, and Ancel keys. Am J Physiol Regul Integr Comp Physiol 295: R704–R713. 10.1152/ajpregu.90337.2008 18525009

[bib17] Fischbarg J (2010) Fluid transport across leaky epithelia: Central role of the tight junction and supporting role of aquaporins. Physiol Rev 90: 1271–1290. 10.1152/physrev.00025.2009 20959616

[bib18] Fischer G, Kosinska-Eriksson U, Aponte-Santamaria C, Palmgren M, Geijer C, Hedfalk K, Hohmann S, de Groot BL, Neutze R, Lindkvist-Petersson K (2009) Crystal structure of a yeast aquaporin at 1.15 Å reveals a novel gating mechanism. PLoS Biol 7: e1000130. 10.1371/journal.pbio.1000130 19529756 PMC2688079

[bib19] Frick A, Jarva M, Ekvall M, Uzdavinys P, Nyblom M, Tornroth-Horsefield S (2013a) Mercury increases water permeability of a plant aquaporin through a non-cysteine-related mechanism. Biochem J 454: 491–499. 10.1042/bj20130377 23819815

[bib20] Frick A, Jarva M, Tornroth-Horsefield S (2013b) Structural basis for pH gating of plant aquaporins. FEBS Lett 587: 989–993. 10.1016/j.febslet.2013.02.038 23454640

[bib21] Fu D, Libson A, Miercke LJW, Weitzman C, Nollert P, Krucinski J, Stroud RM (2000) Structure of a glycerol-conducting channel and the basis for its selectivity. Science 290: 481–486. 10.1126/science.290.5491.481 11039922

[bib22] Gonen T, Cheng Y, Sliz P, Hiroaki Y, Fujiyoshi Y, Harrison SC, Walz T (2005) Lipid-protein interactions in double-layered two-dimensional AQP0 crystals. Nature 438: 633–638. 10.1038/nature04321 16319884 PMC1350984

[bib23] Gonen T, Sliz P, Kistler J, Cheng Y, Walz T (2004) Aquaporin-0 membrane junctions reveal the structure of a closed water pore. Nature 429: 193–197. 10.1038/nature02503 15141214

[bib24] Grosell M (2010) The role of the gastrointestinal tract in salt and water balance. In Fish Physiology. Grosell M, Farrell AP, Colin JB (eds.). pp 135–164. San Diego, CA: Academic Press.

[bib25] Guo H, Wei M, Liu Y, Zhu Y, Xu W, Meng L, Wang N, Shao C, Lu S, Gao F, (2017) Molecular cloning and expression analysis of the aqp1aa gene in half-smooth tongue sole (Cynoglossus semilaevis). PLoS One 12: e0175033. 10.1371/journal.pone.0175033 28380032 PMC5381947

[bib26] Hanamura K, Washburn HR, Sheffler-Collins SI, Xia NL, Henderson N, Tillu DV, Hassler S, Spellman DS, Zhang G, Neubert TA, (2017) Extracellular phosphorylation of a receptor tyrosine kinase controls synaptic localization of NMDA receptors and regulates pathological pain. PLoS Biol 15: e2002457. 10.1371/journal.pbio.2002457 28719605 PMC5515392

[bib27] Hareza A, Bakun M, Swiderska B, Dudkiewicz M, Koscielny A, Bajur A, Jaworski J, Dadlez M, Pawlowski K (2018) Phosphoproteomic insights into processes influenced by the kinase-like protein DIA1/C3orf58. PeerJ 6: e4599. 10.7717/peerj.4599 29666759 PMC5896498

[bib28] Hedfalk K, Tornroth-Horsefield S, Nyblom M, Johanson U, Kjellbom P, Neutze R (2006) Aquaporin gating. Curr Opin Struct Biol 16: 447–456. 10.1016/j.sbi.2006.06.009 16837191

[bib29] Ho JD, Yeh R, Sandstrom A, Chorny I, Harries WEC, Robbins RA, Miercke LJW, Stroud RM (2009) Crystal structure of human aquaporin 4 at 1.8 Å and its mechanism of conductance. Proc Natl Acad Sci U S A 106: 7437–7442. 10.1073/pnas.0902725106 19383790 PMC2678640

[bib30] Horng JL, Chao PL, Chen PY, Shih TH, Lin LY (2015) Aquaporin 1 is involved in acid secretion by ionocytes of zebrafish embryos through facilitating CO2 transport. PLoS One 10: e0136440. 10.1371/journal.pone.0136440 26287615 PMC4546062

[bib31] Ip YK, Soh MML, Chen XL, Ong JLY, Chng YR, Ching B, Wong WP, Lam SH, Chew SF (2013) Molecular characterization of branchial aquaporin 1aa and effects of seawater acclimation, emersion or ammonia exposure on its mRNA expression in the gills, gut, kidney and skin of the freshwater climbing perch, Anabas testudineus. PLoS One 8: e61163. 10.1371/journal.pone.0061163 23593418 PMC3621907

[bib60] Isaksson S, Watkins EB, Browning KL, Kjellerup Lind T, Cárdenas M, Hedfalk K, Höök F, Andersson M (2017) Protein-containing lipid bilayers intercalated with size-matched mesoporous silica thin films. Nano Lett 17: 476–485. 10.1021/acs.nanolett.6b04493 28073257

[bib32] Ishibashi K, Sasaki S, Kondo S (1997) Aquaporin water channels in mammals. Clin Exp Nephrol 1: 247–253. 10.1007/bf02480636 19085041

[bib33] Kinoshita M, Era T, Jakt LM, Nishikawa SI (2009) The novel protein kinase Vlk is essential for stromal function of mesenchymal cells. Development 136: 2069–2079. 10.1242/dev.026435 19465597

[bib34] Kirscht A, Kaptan SS, Bienert GP, Chaumont F, Nissen P, de Groot BL, Kjellbom P, Gourdon P, Johanson U (2016) Crystal structure of an ammonia-permeable aquaporin. PLoS Biol 14: e1002411. 10.1371/journal.pbio.1002411 27028365 PMC4814140

[bib35] Kosinska Eriksson U, Fischer G, Friemann R, Enkavi G, Tajkhorshid E, Neutze R (2013) Subangstrom resolution X-ray structure details aquaporin-water interactions. Science 340: 1346–1349. 10.1126/science.1234306 23766328 PMC4066176

[bib36] Krieger E, Vriend G (2015) New ways to boost molecular dynamics simulations. J Comput Chem 36: 996–1007. 10.1002/jcc.23899 25824339 PMC6680170

[bib37] Krieger E, Vriend G (2014) YASARA View - molecular graphics for all devices - from smartphones to workstations. Bioinformatics 30: 2981–2982. 10.1093/bioinformatics/btu426 24996895 PMC4184264

[bib38] Lema SC, Carvalho PG, Egelston JN, Kelly JT, McCormick SD (2018) Dynamics of gene expression responses for ion transport proteins and aquaporins in the gill of a euryhaline pupfish during freshwater and high-salinity acclimation. Physiol Biochem Zool 91: 1148–1171. 10.1086/700432 30334669

[bib39] Leslie AGW (1992) Recent changes to the MOSFLM package for processing film and image plate data. Joint CCP4 + ESF-EAMCB Newsl Protein Crystallogr 26.

[bib40] Madsen SS, Engelund MB, Cutler CP (2015) Water transport and functional dynamics of aquaporins in osmoregulatory organs of fishes. Biol Bull 229: 70–92. 10.1086/bblv229n1p70 26338871

[bib41] Maier JA, Martinez C, Kasavajhala K, Wickstrom L, Hauser KE, Simmerling C (2015) ff14SB: Improving the accuracy of protein side chain and backbone parameters from ff99SB. J Chem Theory Comput 11: 3696–3713. 10.1021/acs.jctc.5b00255 26574453 PMC4821407

[bib42] Nesverova V, Tornroth-Horsefield S (2019) Phosphorylation-dependent regulation of mammalian aquaporins. Cells 8: 82. 10.3390/cells8020082 PMC6406877 30678081

[bib43] Newby ZER, O’Connell J III, Robles-Colmenares Y, Khademi S, Miercke LJ, Stroud RM (2008) Crystal structure of the aquaglyceroporin PfAQP from the malarial parasite Plasmodium falciparum. Nat Struct Mol Biol 15: 619–625. 10.1038/nsmb.1431 18500352 PMC2568999

[bib44] Nordzieke DE, Medrano-Fernandez I (2018) The plasma membrane: A platform for intra- and intercellular redox signaling. Antioxidants (Basel) 7: 168. 10.3390/antiox7110168 PMC6262572 30463362

[bib45] Nyblom M, Frick A, Wang Y, Ekvall M, Hallgren K, Hedfalk K, Neutze R, Tajkhorshid E, Tornroth-Horsefield S (2009) Structural and functional analysis of SoPIP2;1 mutants adds insight into plant aquaporin gating. J Mol Biol 387: 653–668. 10.1016/j.jmb.2009.01.065 19302796

[bib61] Öberg F, Sjöhamn J, Conner MT, Bill RM, Hedfalk K (2011a) Improving recombinant eukaryotic membrane protein yields in Pichia pastoris: The importance of codon optimization and clone selection. Mol Membr Biol 28: 398–411. 10.3109/09687688.2011.602219 21770695

[bib62] Öberg F, Sjöhamn J, Fischer G, Moberg A, Pedersen A, Neutze R, Hedfalk K (2011b) Glycosylation increases the thermostability of human aquaporin 10 protein. J Biol Chem 286: 31915–23. 10.1074/jbc.M111.242677 21733844 PMC3173105

[bib46] Ozu M, Galizia L, Acuna C, Amodeo G (2018) Aquaporins: More than functional monomers in a tetrameric arrangement. Cells 7: 209. 10.3390/cells7110209 PMC6262540 30423856

[bib47] Reichow SL, Clemens DM, Freites JA, Nemeth-Cahalan KL, Heyden M, Tobias DJ, Hall JE, Gonen T (2013) Allosteric mechanism of water-channel gating by Ca-calmodulin. Nature Struct Mol Biol 20: 1085–1092. 10.1038/nsmb.2630 23893133 PMC3766450

[bib48] Roche JV, Survery S, Kreida S, Nesverova V, Ampah-Korsah H, Gourdon M, Deen PMT, Tornroth-Horsefield S (2017) Phosphorylation of human aquaporin 2 (AQP2) allosterically controls its interaction with the lysosomal trafficking protein LIP5. J Biol Chem 292: 14636–14648. 10.1074/jbc.m117.788364 28710278 PMC5582854

[bib63] Romanos MA, Scorer CA, Clare JJ (1992) Foreign gene expression in yeast: A review. Yeast 8: 423–488. 10.1002/yea.320080602 1502852

[bib49] Smart OS, Goodfellow JM, Wallace BA (1993) The pore dimensions of gramicidin A. Biophys J 65: 2455–2460. 10.1016/s0006-3495(93)81293-1 7508762 PMC1225986

[bib50] Sui H, Han BG, Lee JK, Walian P, Jap BK (2001) Structural basis of water-specific transport through the AQP1 water channel. Nature 414: 872–878. 10.1038/414872a 11780053

[bib51] Sundell KS, Sundh H (2012) Intestinal fluid absorption in anadromous salmonids: Importance of tight junctions and aquaporins. Front Physiol 3: 388. 10.3389/fphys.2012.00388 23060812 PMC3460234

[bib52] Svensson O, Malbet-Monaco S, Popov A, Nurizzo D, Bowler MW (2015) Fully automatic characterization and data collection from crystals of biological macromolecules. Acta Crystallogr D Biol Crystallogr 71: 1757–1767. 10.1107/s1399004715011918 26249356 PMC4528805

[bib53] Tagliabracci VS, Wiley SE, Guo X, Kinch LN, Durrant E, Wen J, Xiao J, Cui J, Nguyen KB, Engel JL, (2015) A single kinase generates the majority of the secreted phosphoproteome. Cell 161: 1619–1632. 10.1016/j.cell.2015.05.028 26091039 PMC4963185

[bib54] Talbot K, Kwong RWM, Gilmour KM, Perry SF (2015) The water channel aquaporin-1a1 facilitates movement of CO(2) and ammonia in zebrafish (Danio rerio) larvae. J Exp Biol 218: 3931–3940. 10.1242/jeb.129759 26677259

[bib55] Tay YL, Loong AM, Hiong KC, Lee SJ, Tng YYM, Wee NLJ, Lee SML, Wong WP, Chew SF, Wilson JM, (2006) Active ammonia transport and excretory nitrogen metabolism in the climbing perch, Anabas testudineus, during 4 days of emersion or 10 minutes of forced exercise on land. J Exp Biol 209: 4475–4489. 10.1242/jeb.02557 17079718

[bib56] Tingaud-Sequeira A, Chauvigne F, Fabra M, Lozano J, Raldua D, Cerda J (2008) Structural and functional divergence of two fish aquaporin-1 water channels following teleost-specific gene duplication. BMC Evol Biol 8: 259. 10.1186/1471-2148-8-259 18811940 PMC2564943

[bib57] Tornroth-Horsefield S, Hedfalk K, Fischer G, Lindkvist-Petersson K, Neutze R (2010) Structural insights into eukaryotic aquaporin regulation. FEBS Lett 584: 2580–2588. 10.1016/j.febslet.2010.04.037 20416297

[bib58] Tornroth-Horsefield S, Wang Y, Hedfalk K, Johanson U, Karlsson M, Tajkhorshid E, Neutze R, Kjellbom P (2006) Structural mechanism of plant aquaporin gating. Nature 439: 688–694. 10.1038/nature04316 16340961

[bib59] Wang C, Xu H, Lin S, Deng W, Zhou J, Zhang Y, Shi Y, Peng D, Xue Y (2020) GPS 5.0: An update on the prediction of kinase-specific phosphorylation sites in proteins. Genomics Proteomics Bioinformatics 18: 72–80. 10.1016/j.gpb.2020.01.001 32200042 PMC7393560

