# Supplementary material for: High-resolution structure of a fish aquaporin reveals a novel extracellular fold
Source: Life Sci Alliance. 2022 Oct 13;5(12):e202201491. doi: 10.26508/lsa.202201491 (PMC9559756; doi:10.26508/lsa.202201491)
Supplement: Supplementary file 1 [file LSA-2022-01491_TableS1.docx]

**Table** **S1. Data collection and refinement statistics of cpAQP1aa**. Values in parentheses are for the highest resolution shell.

| **Crystal** | **Form A (pH 7.8)** | **Form B (pH 6.5)** |
| --- | --- | --- |
| **Condition** | 0.1 M Tris-HCl (pH 7.8), 5% (w/v) γ-PGA (Na+ form, LM), 30% (v/v) PEG400 and 3% (w/v) D-Sorbitol | 0.3 M Lithium sulfate, 0.1 M ADA (pH 6.5), 30% (v/v) PEG400 and 3% (w/v) Dextran sulfate sodium salt (Mr 5,000) |
| **Data collection** |  |  |
| Space group | P42_1_2 | C222_1_ |
| Unit Cell parameters |  |  |
| a, b, c (Å) | 80.06 80.06 95.28 | 113.670 178.100 177.900 |
| Resolution (Å) | 95.28-1.90 (2.00-1.90) | 95.82-3.46 (3.65-3.46) |
| R_merge_ | 0.108 (0.657) | 0.177 (0.832) |
| Mean I/σ(I) | 15.1 (4.0) | 4.5 (1.3) |
| Completeness (%) | 100 (100) | 98.8 (97.9) |
| Redundancy | 12.2 (12.1) | 4.5 (4.3) |
| Number of unique reflections | 25108 (3588) | 23598 (3367) |
| Wilson B-factor (Å^2^) | 25.19 | 89.59 |
| **Refinement** |  |  |
| Resolution (Å) | 56.61-1.9 (1.97-1.9) | 84.36-3.46 (3.58-3.46) |
| R_work_/R_free_ | 0.18/0.20 | 0.27/0.30 |
| Number of protein atoms | 1594 | 6438 |
| Average B-factors(Å^2^) | 36.39 | 119.41 |
| Ramachandran plot statistics (%) |  |  |
| Favored region | 95.28 | 90.4 |
| Allowed region | 3.77 | 8.1 |
| Disallowed region | 0.94 | 1.5 |
| RMSD bond lengths (Å) | 0.014 | 0.003 |
| RMSD bond angles(º) | 1.171 | 0.704 |
