# Supplementary material for: High-resolution structure of a fish aquaporin reveals a novel extracellular fold
Source: Life Sci Alliance. 2022 Oct 13;5(12):e202201491. doi: 10.26508/lsa.202201491 (PMC9559756; doi:10.26508/lsa.202201491)
Supplement: Supplementary file 2 [file LSA-2022-01491_TableS2.docx]

**Table** **S2. Water flow for cpAQP1aa wild type and mutants.** The initial rates for water flow are calculated for proteoliposomes with reconstituted cpAQP1aa, n=6-31 +/- standard deviation, derived from at least two different liposome preps. The initial rates are also normalized to the relative amount of reconstituted protein based on the intensity of the immunoblot signal quantified for each protein target (n≥2), given as a normalization factor relative cpAQP1aa-FL. Poor reconstitution efficiency is observed for Y107A and L117A which correlates with low production levels of those proteins when recombinantly produced in *P. pastoris.* The mutant Y107S was made to mimic this position in human AQP1, and the effect was very similar to wild type.

|  | **initial** | **st dev** | **averaged** | **liposome** | **normalization** | **st dev** | **immunoblots** | **initial rate** | **st dev** |
| --- | --- | --- | --- | --- | --- | --- | --- | --- | --- |
|  | **rate** |  | **curves** | **preps** | **factor** |  |  | **normalized** |  |
|  | **s-1** |  | **n** | **n** |  |  | **n** | **s-1** |  |
| **empty liposome** | 9.54 | 3.49 | 31 | 11 |  |  |  | 9.5 | 3.5 |
| **hAQP4** | 95.13 | 31.76 | 12 | 4 | 1.00 | 0.12 | 2 | 95.1 | 43.1 |
| **cpAQP1aa-243** | 39.97 | 11.92 | 9 | 3 |  |  |  |  |  |
| **cpAqp1aa-FL** | **34.23** | **13.65** | 24 | 8 | **1.00** | **0** | **6** | **34.2** | **13.7** |
| **cpAqp1aa-L117A** | 7.49 | 0.73 | 6 | 2 | 4.05 | 1.82 | 2 | 30.3 | 16.6 |
| **cpAqp1aa-Y107A** | 23.26 | 8.14 | 6 | 2 | 3.18 | 0.19 | 2 | 74.0 | 30.3 |
| **cpAqp1aa-Y107S** | 39.63 | 6.68 | 6 | 2 | 1.32 | 0.24 | 2 | 52.3 | 18.3 |
| **cpAqp1aa-T38A** | 51.01 | 15.76 | 6 | 2 | 1.08 | 0.10 | 2 | 55.1 | 22.1 |
| **cpAqp1aa-T38E** | 64.38 | 19.50 | 6 | 2 | 1.07 | 0.06 | 2 | 68.9 | 24.7 |
| **cpAqp1aa-Y107E** | 33.89 | 2.00 | 6 | 2 | 0.95 | 0.19 | 4 | 32.2 | 8.3 |
